# Nicotinamide riboside enhances adoptive T cell therapy by promoting memory differentiation and metabolic fitness

**DOI:** 10.1016/j.ymthe.2026.05.026

**Published:** 2026-06-02

**Authors:** Nengzhi Pang, Jinping He, Lei Pei, Ying Xiao, Yingying Gu, Cheng Zhi, Xuye Lai, Mingtao Chen, Qiyao Xiao, Xueqian Wu, Zhenfeng Zhang, Lili Yang

**Affiliations:** 1 Department of Nutrition, School of Public Health, Sun Yat-sen University, Guangzhou, Guangdong 510275, China; 2 Radiology Center, Guangzhou Key Laboratory for Research and Development of Nano-Biomedical Technology for Diagnosis and Therapy, Guangdong Provincial Education Department Key Laboratory of Nano-Immunoregulation Tumour Microenvironment, Central Laboratory, the Second Affiliated Hospital, Guangzhou Medical University, Guangzhou, Guangdong 510260, China; 3 Key Laboratory of Tropical Translational Medicine of Ministry of Education, International Collaborative Research Center for the Development and Utilization of Tropical Food for Special Medical Purpose, School of Public Health, Hainan Medical University, Haikou, Hainan 571199, China; 4 Hainan Academy of Medical Sciences, Hainan Medical University, Haikou, Hainan 571199, China; 5 Department of Ultrasound, Guangdong Women and Children Hospital, Guangzhou, Guangdong 510000, China; 6 Department of Pathology, the Second Affiliated Hospital of Guangzhou Medical University, Guangzhou, Guangdong 510260, China

**Keywords:** CD8^+^ T cell, adoptive T cell therapy, central memory T cell, anti-tumor capacity, nicotinamide riboside, hepatocellular carcinoma, HCC

## Abstract

Enhancing the efficacy of adoptively transferred T cells is essential for successful immunotherapy. The cytotoxic activity of these T cells is closely related to their mitochondrial function, which plays a critical role in T cell differentiation. This study explored the potential of nicotinamide riboside (NR), known to enhance mitochondrial function, to improve the efficacy of adoptively transferred CD8^+^ T cells against hepatocellular carcinoma (HCC). In subcutaneous and metastatic HCC tumor models, NR treatment significantly improved the effectiveness of adoptive T cell therapy (ACT). NR promoted the differentiation of CD44^hi^CD62L^hi^ central memory T cells (T_CM_) and upregulated memory-associated genes (*Tcf7*, *Ccr7*, and *Foxo1*). Upon restimulation, NR-treated CD8^+^ T cells exhibited strong recall responses, as evidenced by metabolic reprogramming and increased cytokine production. Furthermore, RNA sequencing revealed an increased expression of *Foxo1* and its target genes. Inhibition of Foxo1 partially reversed the beneficial effects of NR on mitochondrial fitness and cytotoxic function, suggesting a role for Foxo1-associated pathways in mediating these effects. In summary, NR improved mitochondrial fitness and promoted T_CM_ differentiation, in part through a transcriptional program associated with Foxo1, thereby improving the efficacy of ACT. These findings identify NR as a promising and translatable metabolic adjuvant for HCC immunotherapy.

## Graphical abstract

**Figure:**
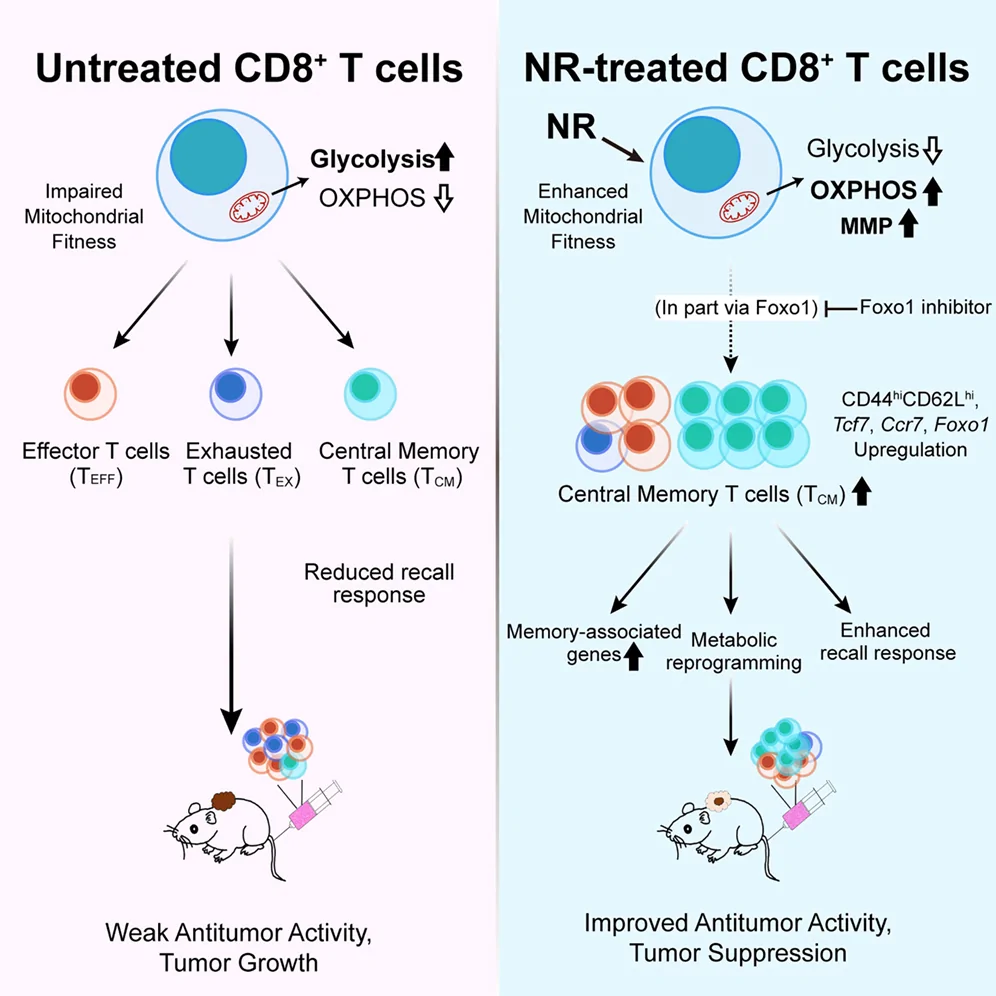

## Teaser

Nicotinamide riboside (NR) enhances adoptive T cell therapy by improving mitochondrial fitness and promoting central memory T cell differentiation. NR-treated T cells exhibit stronger recall responses and anti-tumor activity in hepatocellular carcinoma models, supporting NR as a practical metabolic adjuvant to improve the efficacy of adoptive cell therapies.

## Introduction

Cytotoxic T lymphocytes (CTLs), also known as CD8^+^ T cells, are crucial in recognizing and eliminating tumor cells within the tumor microenvironment. Studies have indicated that while effector T cells (T_EFF_) are fundamentally responsible for tumor clearance, transferring less-differentiated T cells, such as stem cell memory T cells (T_SCM_) and central memory T cells (T_CM_), has been shown to confer enhanced anti-tumor efficiency *in vivo*.^1^^,^^2^^,^^3^ T cell differentiation is accompanied by metabolic reprogramming, which in turn affects T cell phenotype and function.^4^^,^^5^ Notably, naive and memory T cells predominantly rely on oxidative phosphorylation (OXPHOS), while T_EFF_ increase aerobic glycolysis to support their rapid growth and energy requirements.^6^ This metabolic reprogramming is considered a crucial regulator of T cells, including regulating T cell differentiation, prolonging T cell lifespan, and improving anti-tumor ability.^5^^,^^7^^,^^8^^,^^9^

Nicotinamide adenine dinucleotide (NAD^+^) is a critical coenzyme in energy metabolism and an essential co-substrate for various enzymes involved in multiple cell processes associated with aging, cancer, and metabolic disorders.^10^^,^^11^ Recent studies have shown that NAD^+^ deprivation via overconsumption or pharmacological inhibition of nicotinamide phosphoribosyl transferase (NAMPT) decreases the anti-tumor activity of T cells.^12^^,^^13^ Conversely, restoring NAD^+^ levels can improve T cell responses and functions.^14^^,^^15^ Nicotinamide riboside (NR), an excellent NAD^+^ precursor, is a naturally occurring form of vitamin B_3_ found in dietary sources such as milk.^16^^,^^17^ Current studies have confirmed that NR supplementation markedly improves mitochondrial function and enhances energy metabolism in multiple cells.^18^^,^^19^^,^^20^ However, the specific effects of NR on T cell differentiation and function remain to be fully elucidated. Our prior research, alongside recent findings, demonstrated that NR effectively suppressed tumor growth in immunocompetent mice with hepatocellular carcinoma (HCC) or melanoma. However, this beneficial effect was abolished in the absence of T cells.^21^^,^^22^ These findings prompted us to investigate the impact of NR treatment on the efficacy of adoptive T cell therapy (ACT).

In this study, we conducted both *in vivo* and *in vitro* NR treatments to evaluate their impact on CD8^+^ T cells. We utilized glypican-3 (GPC3)-specific chimeric antigen receptor (CAR)-T cells, a common and potent ACT targeting HCC, and OT-I CD8^+^ T cells to assess their cytotoxic efficacy against tumor cells expressing either GPC3 or ovalbumin (OVA). To explore the mechanisms involved, we performed RNA sequencing (RNA-seq) to identify key signaling pathways and used specific inhibitors to confirm their roles in NR-mediated effects in CD8^+^ T cells.

## Results

### Oral NR treatment enhances the therapeutic efficacy of anti-GPC3 CAR-T cells *in vivo*

Based on our previous finding that NR suppressed tumor growth in immunocompetent C57BL/6J mice but not in T cell-deficient mice,^21^ we further investigated CD8^+^ T cell infiltration in tumor tissues from immunocompetent mice. Immunohistochemistry (IHC) and western blot analyses demonstrated increased infiltration of CD8^+^ T cells in tumor tissues from NR-treated mice (Figure S1). These results led us to hypothesize that NR exerts its anti-tumor effect by enhancing CD8^+^ T cell function.

To test this hypothesis, we generated third-generation anti-GPC3 CAR-T cells with a lentiviral vector encoding a GPC3-specific CAR sequence (Figure S2). ACT with anti-GPC3 CAR-T cells ± daily oral NR supplementation was applied to two HCC tumor models in immunodeficient mice, allowing us to determine whether NR boosts the function of transferred T cells. In the subcutaneous tumor model (Figure 1A), ACT combined with oral NR significantly suppressed tumor growth compared to ACT alone (Figures 1B and 1C). However, oral NR supplementation alone did not noticeably suppress tumor growth in immunodeficient mice, consistent with our previous study.^21^ Similar results were observed in the metastatic tumor model (Figure 1D). We tracked tumor progression with *in vivo* bioluminescent imaging. No initial differences were found among groups, but significantly lower bioluminescence signals were observed in mice receiving anti-GPC3 CAR-T cells than in non-ACT-treated mice by days 21 and 35 (Figure 1E). Mice treated with combined therapy with ACT and NR had significantly lower bioluminescence signals than those receiving anti-GPC3 CAR-T cells alone (Figure 1F). Moreover, ACT treatment significantly prolonged the survival time of tumor-bearing mice compared with non-ACT-treated mice, especially for those also receiving NR supplementation (Figure 1G). Taken together, these results demonstrate that the anti-tumor effect of NR depends on the presence of T cells and that NR supplementation enhances T cell function *in vivo*, as evidenced by the improved efficacy of CAR-T cells when combined with NR. However, it remains unclear whether NR acts directly on CD8^+^ T cells to exert this effect.

**Figure:**
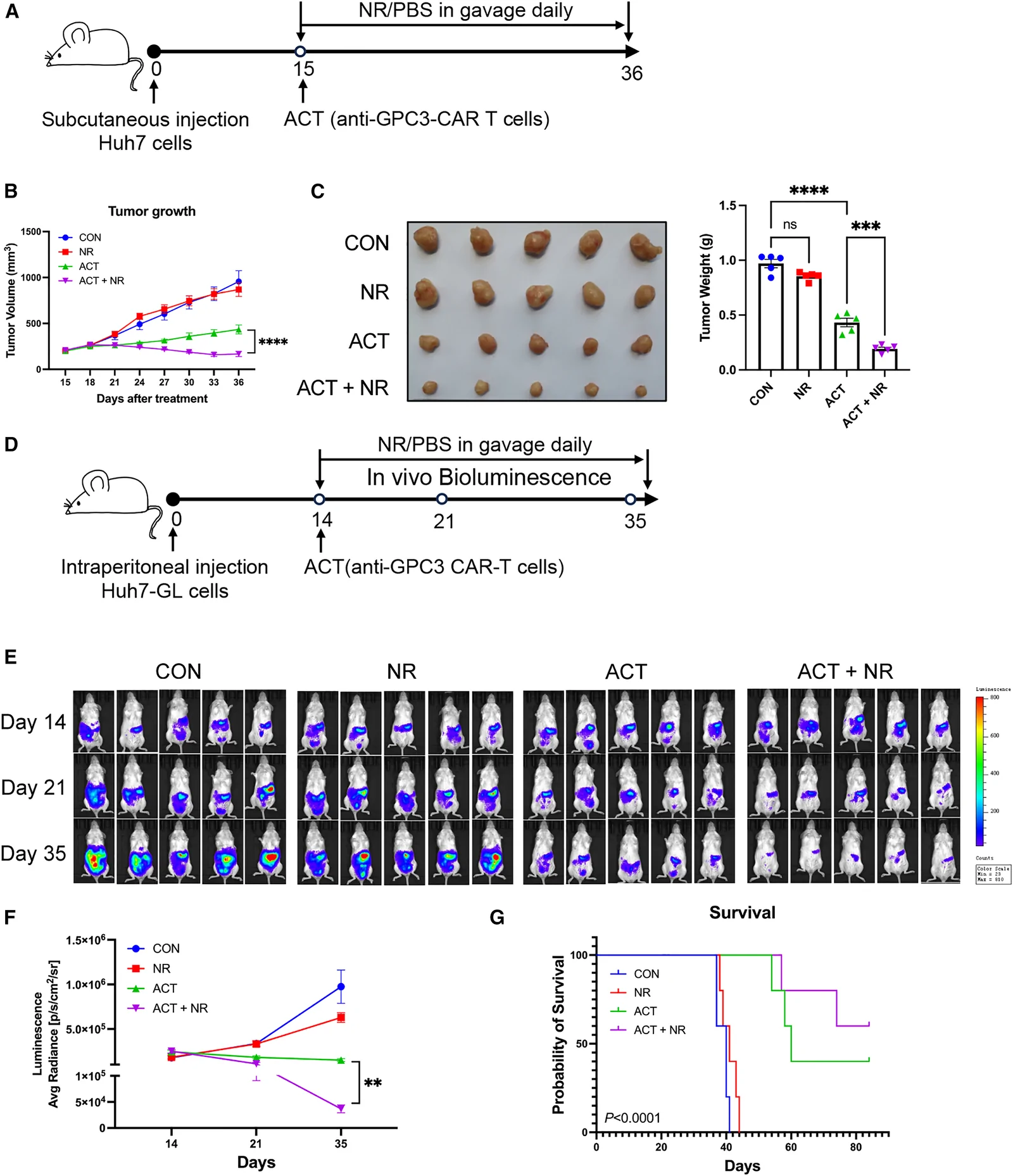
1. NR enhanced the therapeutic efficiency of anti-GPC3 CAR-T cells *in vivo* (A) Schematic of the subcutaneous xenograft tumor model with anti-GPC3 CAR-T cell therapy in NSG mice. (B) Subcutaneous tumor growth of NSG mice after adoptive anti-GPC3 CAR-T cell therapy. (C) Tumor images and weight of NSG mice. (D) Schematic of the metastatic tumor model with anti-GPC3 CAR-T cell therapy in NSG mice. (E and F) *In vivo* bioluminescence images and quantification of each group on days 14, 21, and 35. (G) Survival percentage of NSG mice in 4 groups. ACT, adoptive T cell therapy. *n* = 5/group; ∗∗*p* < 0.01, ∗∗∗*p* < 0.001, and ∗∗∗∗*p* < 0.0001; *p* values were calculated using two-way repeated-measures (RM) ANOVA (B and F), one-way ANOVA and Tukey’s multiple comparisons test (C), and a log rank test for trend (G).

### *In vitro* NR treatment improves the anti-tumor effect of OT-Ι CD8^+^ T cells

To address this, we isolated primary CD8^+^ T cells from the spleen of OT-Ι mice and activated them *in vitro*, with or without NR treatment. Given that NR, a potent precursor of NAD^+^, boosts intracellular NAD^+^ levels and influences mitochondrial energy metabolism, both of which are crucial for T cell differentiation and function, we hypothesized that NR could improve the anti-tumor ability of CD8^+^ T cells by regulating their differentiation. Through a phenotypic analysis of T cell subpopulations in the OT-I CD8^+^ T cells, we found that the proportion of CD44^hi^CD62L^hi^ T_CM_ increased when T cells were treated with NR at 500 or 1,000 μmol/L (Figure S3A). The frequency of T_CM_ significantly increased after 7 days of NR treatment (Figure S3B). Treatment of CD8^+^ T cells with 500 μmol/L of NR for 7 days was applied in our subsequent experiments (Figure 2A).

**Figure:**
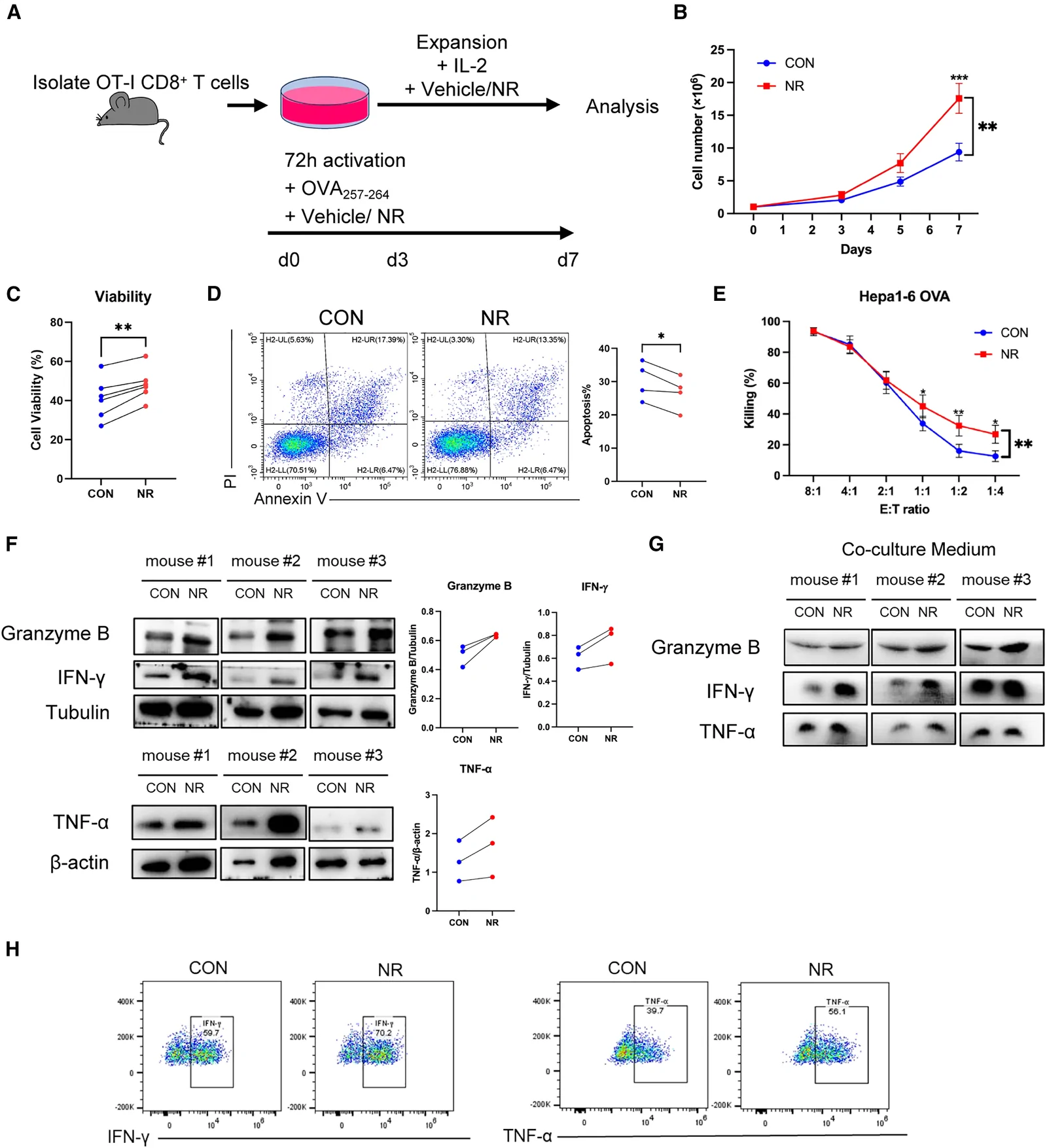
2. NR-treated CD8^+^ T cells showed enhanced anti-tumor ability *in vitro* (A) Schematic of isolation, stimulation, and expansion of mouse primary CD8^+^ T cells. (B) The efficiency of *in vitro* expansion of OT-I CD8^+^ T cells with or without NR intervention for 7 days. *n* = 4 independent experiments. (C) Cell viability after 7 days of *in vitro* culture. *n* = 6 independent experiments. (D) Apoptosis detected by flow cytometry with Annexin V and PI staining in T cells (left). Quantification of the percentage of apoptotic cells in each group (right). *n* = 4 independent experiments. (E) *In vitro* killing assay with day-7 OT-I CD8^+^ T cells, varying the effector-to-target (E:T) ratio for 24 h. *n* = 5 independent experiments. (F and G) Western blot of granzyme B, IFN-γ, and TNF-α of suspension cells (F) and medium (G) in the co-culture wells. *n* = 3 independent experiments. (H) Production of IFN-γ and TNF-α in CD8^+^ T cells under tumor cell-conditioned medium (TCM). ∗*p* < 0.05, ∗∗*p* < 0.01, and ∗∗∗*p* < 0.001; *p* values were calculated using paired *t* test (C, D, and G) and two-way RM ANOVA (E).

NR intervention significantly enhanced T cell expansion efficiency (Figure 2B), although the CFSE proliferation assay did not reveal significant differences in proliferation (Figure S3C). NR promoted *ex vivo* T cell expansion by enhancing cell viability and reducing apoptosis (Figures 2C and 2D). Meanwhile, the killing capacity of NR-treated OT-I CD8^+^ T cells to Hepa1-6 OVA cells significantly increased, especially at a lower effector-to-target (E:T) ratio, compared to control (Figure 2E). Additionally, after 7 days of NR treatment, OT-I CD8^+^ T cells produced higher levels of effector cytokines such as interferon-γ (IFN-γ) and tumor necrosis factor-α (TNF-α) in co-culture conditions or tumor cell-conditioned medium (Figures 2F–2H).

Furthermore, we established subcutaneous and metastatic tumor models and transferred OT-I CD8^+^ T cells into tumor-bearing mice to assess the effect of *in vitro* NR treatment on T cell anti-tumor efficacy. Mice in the control (CON) or NR groups received OT-I CD8^+^ T cells without/with NR pretreatment via tail vein injection, while the PBS group received saline vehicle. In the subcutaneous tumor model (Figures 3A andS4A–S4C), NR-pretreated OT-I CD8^+^ T cells slowed subcutaneous tumor growth compared to the PBS group (Figure 3B). Increasing the frequency of injection of ACT with NR-pretreated OT-I CD8^+^ T cells significantly inhibited tumor growth compared to that with non-NR-pretreated cells (Figures 3C, S4D, and S4E). By the end of the experiment, mice in the NR group showed lower bioluminescence signals at the tumor site, indicating slower tumor growth than the CON group (Figure 3D). In the metastatic tumor model, mice treated with NR-pretreated CD8^+^ T cells resulted in less weight loss, a higher survival rate, and lower fluorescence signals compared to non-NR-pretreated CD8^+^ T cells (Figures 3E–3H).

**Figure:**
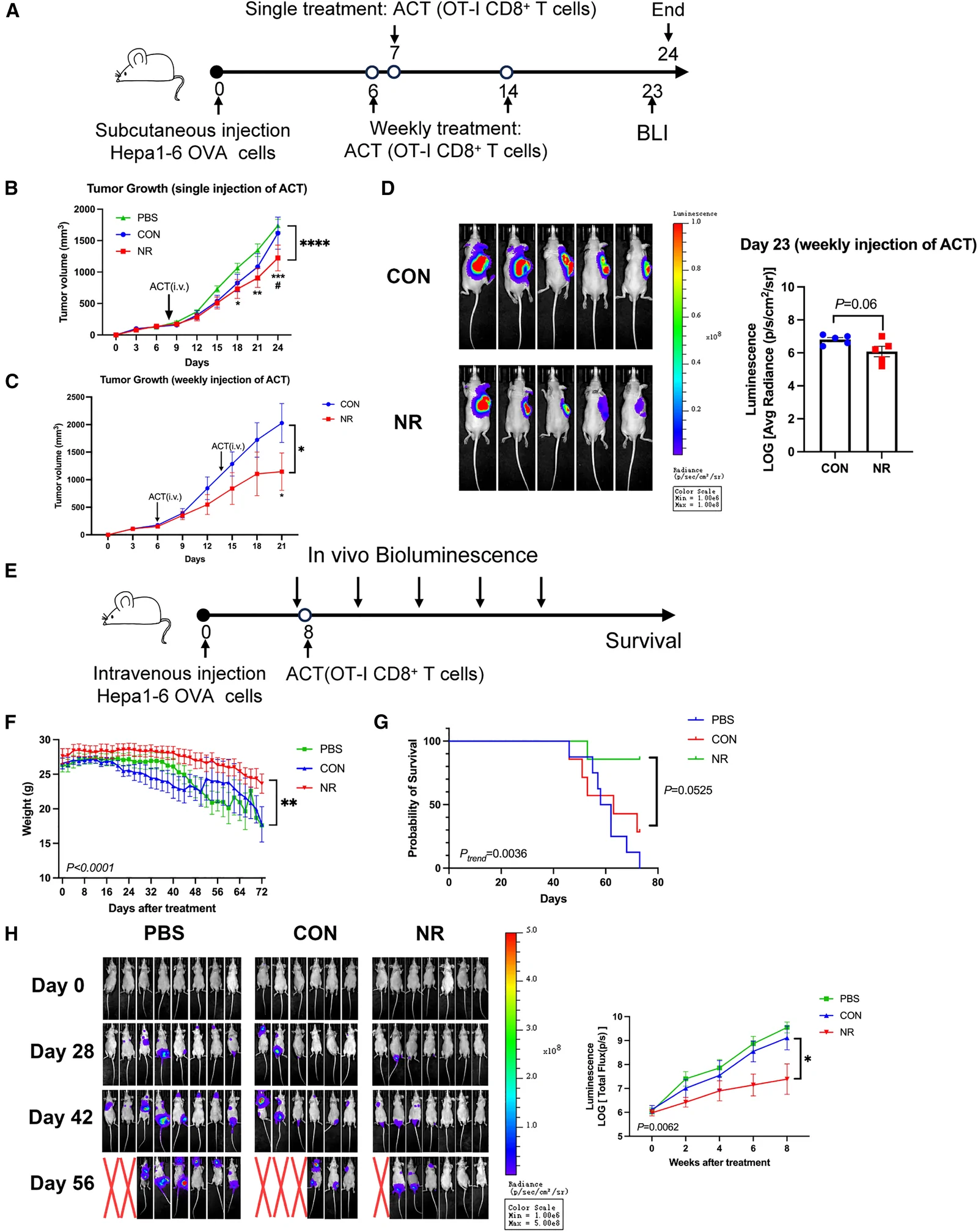
3. NR-treated CD8^+^ T cells showed enhanced anti-tumor ability *in vivo* (A) Schematic of the subcutaneous xenograft tumor model with single or weekly treatment of adoptive T cell therapy. (B) Subcutaneous tumor growth of nude mice in 3 groups. *n* = 6–9/group. (C) Subcutaneous tumor growth of nude mice in the control or NR groups. *n* = 5/group. (D) *In vivo* bioluminescence images and quantification of tumor-bearing mice. (E) Schematic of the metastatic tumor model with adoptive T cell therapy. (F) Body weight of nude mice during the experiment. (G) Survival percentage of nude mice in 3 groups. (H) *In vivo* bioluminescence images of each group. *n* = 6–8/group; ∗*p* < 0.05, ∗∗*p* < 0.01, ∗∗∗*p* < 0.001, and ∗∗∗∗*p* < 0.0001; *p* values were calculated using two-way RM ANOVA (B, C, F, and H) and a log rank test for trend (G). All error bars indicated SEM.

Collectively, both *in vitro* and *in vivo* results indicated that *in vitro* NR treatment improved the anti-tumor activity of CD8^+^ T cells.

### NR promotes the phenotypic differentiation toward T_CM_

Phenotypic differentiation of CD8^+^ T cells was analyzed by flow cytometry after 7 days of NR treatment. NR treatment significantly increased the percentage of CD8^+^ T cells highly expressing both CD44 and CD62L (CD44^hi^CD62L^hi^ T_CM_) (Figure 4A). Memory-related genes, such as *Tcf7*, *Ccr7*, and *Foxo1*, were significantly upregulated in NR-treated OT-I CD8^+^ T cells (Figure 4B). Consistently, transcriptome sequencing results showed that the expression of memory-related genes was significantly upregulated in the NR group (Figure 4C), whereas those associated with effector functions (*Tnf*, *Gzmb*, etc.) were downregulated (Figure 4D). Additionally, NR-treated OT-I CD8^+^ T cells exhibited increased production of perforin and granzyme B in response to secondary stimulation, suggesting a stronger recall response (Figures 4E–4G and S5A–S5C). Consistently, upon secondary stimulation with tumor cells, NR-treated CAR-T cells demonstrated significantly improved viability, diminished exhaustion phenotype, and potentiated effector function (Figures S5D–S5G). These results indicated that NR could promote T_CM_ differentiation.

**Figure:**
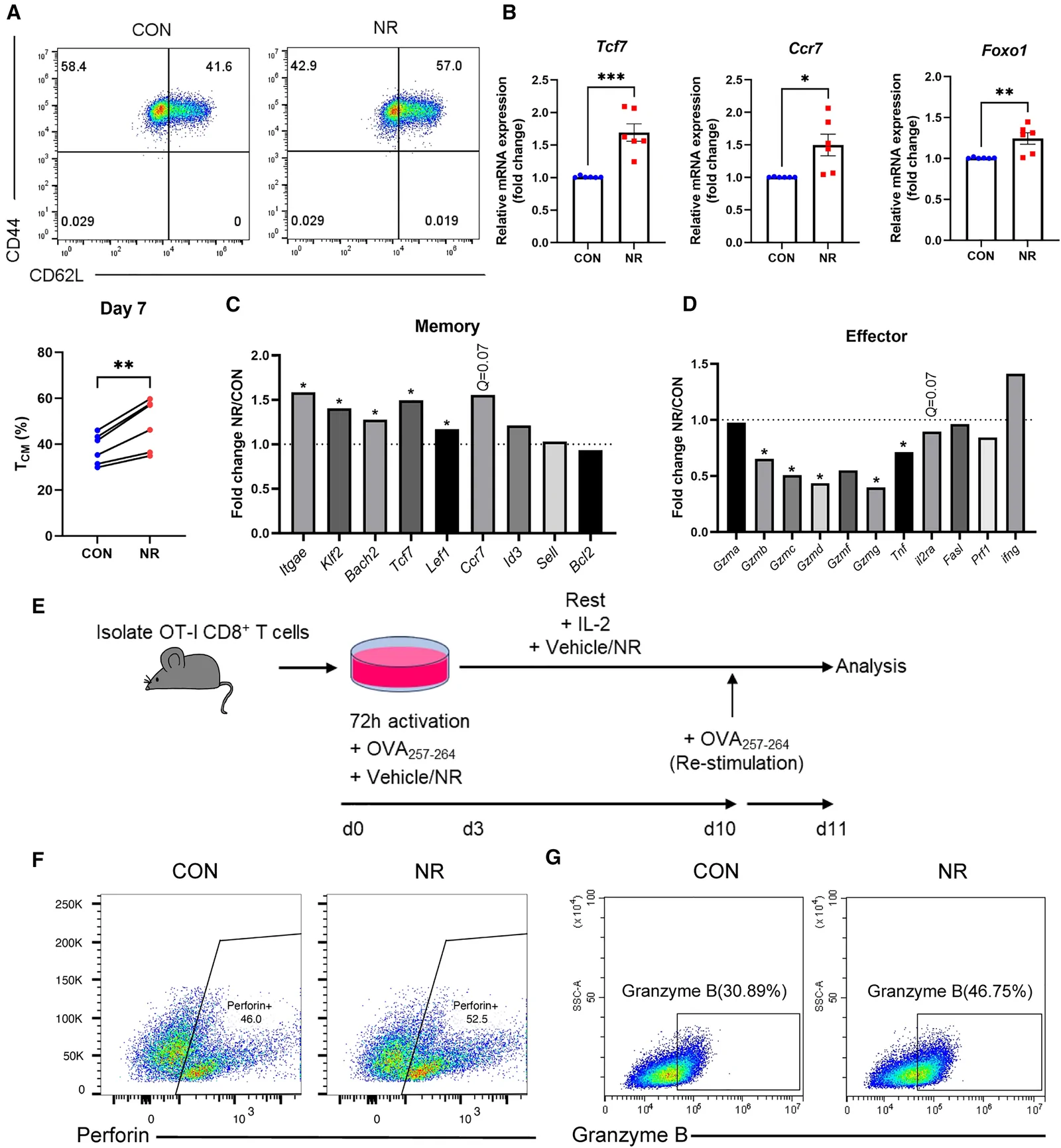
4. NR improved the generation of central memory T cells (A) Frequency of central memory T cells (CD44^hi^CD62L^hi^) after 7 days of NR treatment. *n* = 6 independent experiments. Flow cytometry (FC) data were representative of six independent experiments. (B) Quantitative PCR results of memory-related gene expression in day-7 OT-I CD8^+^ T cells. *n* = 6 independent experiments. (C and D) Genes associated with memory and effector expression in day-7 OT-I CD8^+^ T cells. The asterisk indicates differentially expressed genes (DEGs). (E) Schematic of restimulation of mouse primary CD8^+^ T cells. (F and G) Production of perforin and granzyme B in CD8^+^ T cells upon SIINFEKL peptide restimulation. FC data were representative of four independent experiments. ∗*p* < 0.05, ∗∗*p* < 0.01, and ∗∗∗*p* < 0.001; *p* values were calculated using a paired *t* test (A) and an unpaired *t* test (B). All error bars indicated SEM.

### NR improves the mitochondrial fitness of OT-I CD8^+^ T cells

It has been reported that T cell differentiation is closely linked to mitochondrial function, and NR has been confirmed to significantly improve mitochondrial function in various tissues and cells.^19^^,^^23^^,^^24^^,^^25^ Therefore, we analyzed the effect of NR intervention on mitochondrial function in CD8^+^ T cells. Considering that NR is an excellent NAD^+^ precursor, we first evaluated the intracellular NAD^+^ levels in OT-I CD8^+^ T cells. After 7 days of NR treatment, total NAD^+^/NADH and NAD^+^ levels significantly increased in OT-I CD8^+^ T cells, while NADH levels and NAD^+^/NADH ratios remained unchanged (Figures 5A and S6A). Mitochondrial membrane potential (MMP) was analyzed using JC-1 staining. NR treatment resulted in a significant increase in the red/green fluorescence intensity ratio compared to the CON group (Figure 5B), indicating fewer depolarized mitochondria in CD8^+^ T cells. Confocal laser scanning microscopy (CLSM) images showed the same results (Figure 5C). Additionally, NR-treated CD8^+^ T cells exhibited significantly reduced levels of mitochondrial reactive oxygen species (ROS) and increased expression of the mitochondrial proteins TOM20 and PGC1α (Figures 5D–5F). These findings indicated that NR intervention could sustain healthy mitochondria in CD8^+^ T cells.

**Figure:**
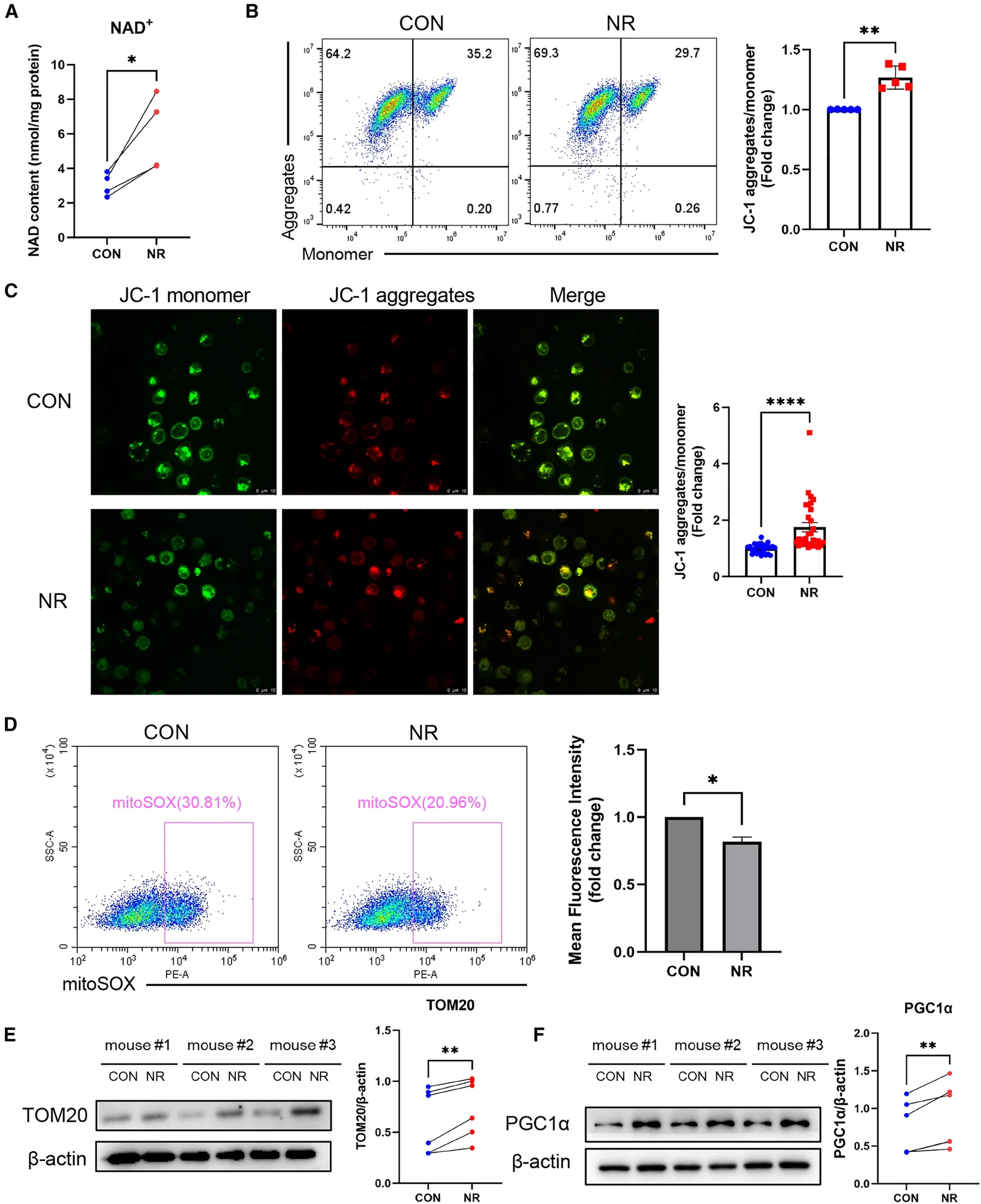
5. NR improved mitochondrial health and function in OT-I CD8^+^ T cells (A) The level of NAD^+^ in T cells. *n* = 4 independent experiments. (B) Flow cytometry (FC) analysis with JC-1 dye. The image on the right is a quantification of the ratio of median fluorescence intensity (MFI) of red (aggregates) to that of green (monomer). FC data were representative of five independent experiments. (C) Representative confocal laser scanning microscopy images with JC-1 probe dye. Quantification of confocal images (right) involves the analysis of red and green fluorescence in individual cells in all pictures (*n* = 3 pictures/group). Scale bars represent 10 μm. (D) FC analysis with mitoSOX staining. *n* = 3 independent experiments. FC data were representative of three independent experiments. (E and F) TOM20 (E) and PGC1α (F) expression in T cells. *n* = 6 independent experiments. ∗*p* < 0.05, ∗∗*p* < 0.01, ∗∗∗*p* < 0.001, and ∗∗∗∗*p* < 0.0001; *p* values were calculated using a paired *t* test (A, E, and F) and an unpaired *t* test (B and D). All error bars indicated SEM.

T cell differentiation is accompanied by metabolic reprogramming. After 7 days of NR treatment, OT-I CD8^+^ T cells exhibited an increased oxygen consumption rate (OCR) and decreased extracellular acidification rate (ECAR) (Figures 6A and 6B). These cells showed higher maximal respiration and spare respiratory capacity (SRC), along with reduced basal glycolysis and glycolytic capacity (Figures 6A and 6B). This suggested that NR-treated OT-I CD8^+^ T cells relied more on mitochondrial OXPHOS to produce energy, whereas non-NR-treated T cells were more dependent on glycolysis. Upon restimulation, NR-treated OT-I CD8^+^ T cells showed a significant increase in both ECAR and OCR compared to CON cells (Figures 6C and 6D), indicating enhanced metabolic reprogramming. Concomitantly, these cells exhibited elevated effector cytokine production (Figures 4E–4G and S5A–S5C) and upregulated terminal effector markers, including KLRG1 and T-bet (Figures S6B and S6C). These findings demonstrate that NR-treated CD8^+^ T cells were more rapidly activated than CON cells upon secondary challenge, further supporting the notion that NR promoted the formation of T_CM_ capable of robust recall responses.

**Figure:**
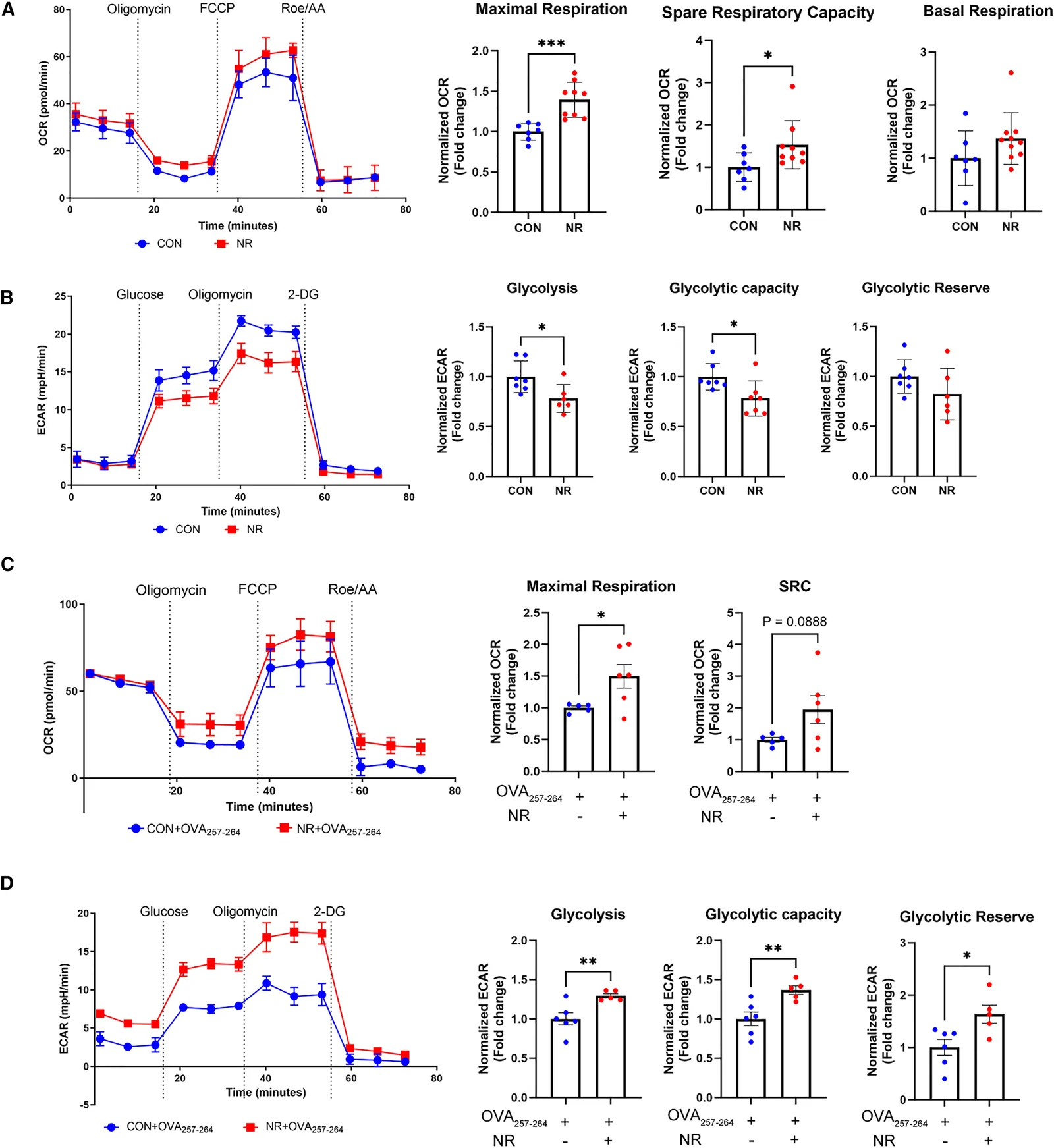
6. NR promotes metabolic reprogramming in OT-I CD8^+^ T cells (A) The Seahorse assay of O_2_ consumption rate (OCR) and quantification. *n* = 3 independent experiments in 3–4 technical replicates. (B) The Seahorse assay of extracellular acidification rate (ECAR) and quantification. *n* = 3 independent experiments in 2–3 technical replicates. (C and D) The Seahorse analysis and quantification of OCR (C) and ECAR (D) upon SIINFEKL peptide restimulation. *n* = 2 independent experiments in 2–3 technical replicates. ns, no significance, ∗*p* < 0.05, ∗∗*p* < 0.01, ∗∗∗*p* < 0.001, and ∗∗∗∗*p* < 0.0001; *p* values were calculated using an unpaired *t* test. All error bars indicated SEM.

### NR activates the Foxo1 signaling pathway to regulate mitochondrial fitness

To explore potential mechanisms by which NR treatment affects the differentiation and function of CD8^+^ T cells, we conducted a comprehensive analysis of RNA-seq data. Both KEGG pathway enrichment analysis and pathway network analysis revealed that the upregulated genes in the NR group were significantly enriched in the Foxo signaling pathway (Figures 7A and S7A). Notably, the *Foxo1* gene, along with its mRNA and protein expression and its target genes, showed significant upregulation in NR-treated OT-I CD8^+^ T cells (Figures S7B, S7C, 4B, and 7B). These findings suggested that Foxo1 may play a crucial role in modulating T cell differentiation induced by NR treatment. To validate this hypothesis, OT-I CD8^+^ T cells were treated with AS1842856, a specific Foxo1 inhibitor (Figure 7C). Foxo1 inhibition resulted in a significant decrease in the killing ability of CD8^+^ T cells against HCC cells, which could not be restored by NR treatment (Figure 7D). Meanwhile, the enhancement of T_CM_ differentiation and the improvement in mitochondrial health induced by NR were ameliorated when Foxo1 was inhibited (Figures 7E and 7F). These results suggested a role for Foxo1 in mediating, at least in part, the NR-triggered effects on mitochondrial fitness and enhanced killing capacity of CD8^+^ T cells against HCC cells.

**Figure:**
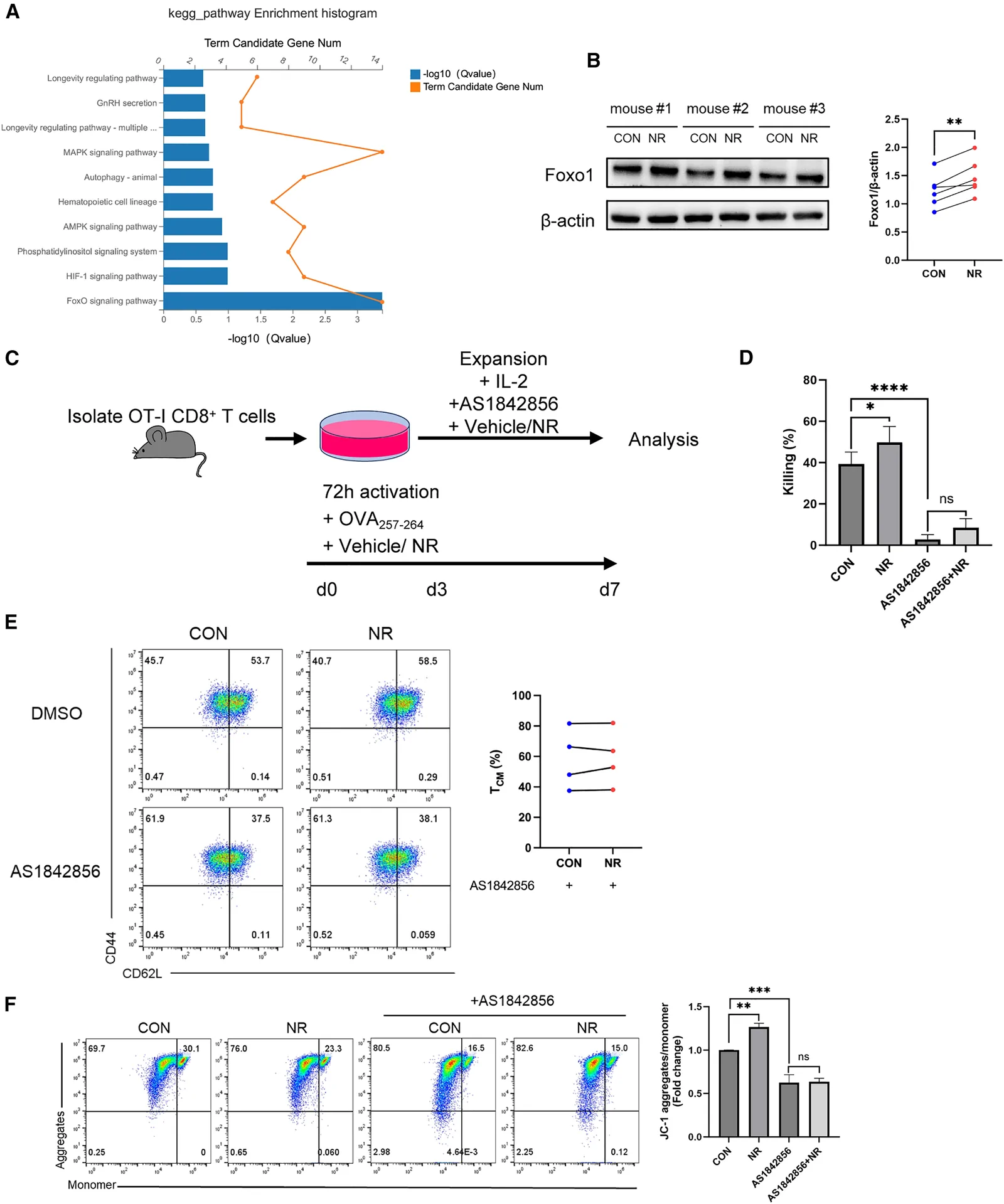
7. NR regulated mitochondrial fitness via activating the Foxo1 signaling pathway (A) KEGG pathway enrichment analysis of upregulated genes in the NR group. (B) Foxo1 protein expression in day-7 OT-I CD8^+^ T cells. *n* = 6 independent experiments. (C) Schematic of Foxo1 inhibition in mouse primary CD8^+^ T cells. (D) *In vitro* killing assay with day-7 OT-I CD8+ T cells at a 1:1 effector-to-target (E:T) ratio for 24 h. *n* = 2 independent experiments in 3–4 technical replicates. (E) Flow cytometry (FC) of T cell differentiation of CD8^+^ T cells treated with 100 nmol/L AS1842856 for 48 h after activation. FC data were representative of four independent experiments. (F) Quantification of FC analysis with JC-1 staining after AS1842856 treatment for 96 h. *n* = 4 independent experiments. ∗*p* < 0.05, ∗∗*p* < 0.01, ∗∗∗*p* < 0.001, and ∗∗∗∗*p* < 0.0001; *p* values were calculated using a paired *t* test (B) and one-way ANOVA and a Tukey’s multiple comparisons test (D and F). All error bars indicated SEM.

## Discussion

Our study elucidates that both administering NR *in vivo* and *in vitro* significantly improves the effectiveness of ACT. This protective effect is associated with improved mitochondrial fitness, promotion of T_CM_ differentiation, and enhanced CD8^+^ T cell killing capacity against liver cancer cells, partly via a Foxo1-associated signaling pathway.

The role of NAD^+^ in tumor progression is still debated. Although numerous studies have emphasized the anti-tumor effects of inhibiting key rate-limiting enzymes in NAD^+^ synthesis, such as NAMPT and NAPRT, across different types of tumors,^26^^,^^27^ recent studies have presented a different perspective, indicating that increasing NAD^+^ levels may suppress tumor progression.^28^^,^^29^^,^^30^ A recent study demonstrated that oral NR supplementation suppressed tumor growth in melanoma-engrafted mice, and this anti-tumor effect was abolished by CD8^+^ T cell depletion.^22^ Similarly, in our previous study, NR showed an anti-tumor effect in immunocompetent mice but not in immunodeficient mice lacking mature T cells.^21^ In this study, we observed increased infiltration of CD8^+^ T cells into tumor tissues of mice receiving oral NR supplementation, suggesting the potential therapeutic significance of NR in cancer treatment by modulating immune response mechanisms.

NAD^+^ plays a critical role in cell metabolism and regulates multiple cell processes. NAD^+^ depletion caused by CD38 overexpression (an NAD^+^-consuming enzyme) or NAMPT inhibitor induces T cell apoptosis and dysfunction.^14^^,^^15^^,^^31^ In contrast, *in vitro* supplementation with NAD^+^ precursors, including nicotinamide (NAM),^13^^,^^32^ nicotinamide mononucleotide (NMN),^33^^,^^34^ and nicotinic acid (NA),^32^ can prevent mitochondrial dysfunction and improve T cell responses. Consistently, in our study, NR, as an NAD^+^ precursor, markedly increased intracellular NAD^+^ levels in CD8^+^ T cells, improving mitochondrial fitness and promoting T_CM_ formation, leading to enhanced anti-tumor ability. While both NR and NMN aided T_CM_ differentiation, NR had a stronger effect (Figure S8). Previous study also showed that boosting NAD^+^ levels with NAM supplementation enhances the anti-tumor activity of anti-CD19 CAR-T cells *in vivo*.^13^ Notably, the latest study showed that NMN, combined with a CD38 inhibitor, can rescue age-associated NAD^+^ decline in CAR-T cells, alleviating mitochondrial dysfunction and enhancing the efficacy of CAR-T therapy.^34^ Our results further support the idea that increasing NAD^+^ can enhance the therapeutic efficacy of ACT.

Metabolic reprogramming has increasingly been recognized as a critical regulator of T cell fate and function.^6^ Numerous studies have demonstrated that improving mitochondrial fitness and function, or inhibiting glycolysis, can promote the generation of memory T cells and improve the survival and function of T cells.^35^^,^^36^^,^^37^ Consistent with previous studies, our results revealed that boosting intracellular NAD^+^ levels with NR supplementation improved mitochondrial fitness and function, evidenced by the increased MMP, PGC1α expression, and OXPHOS. This suggested that the mechanism by which NR promotes T_CM_ differentiation might be related to the improvement of mitochondrial fitness and function.

Previous studies have demonstrated that T_CM_ exhibit high OXPHOS and lower cytokine production.^1^^,^^38^ Upon antigen rechallenge, T_CM_ respond rapidly and subsequently differentiate into T_EFF_, displaying increased energy metabolism, including both OXPHOS and glycolysis, which correlates with enhanced anti-tumor ability.^39^ In our current study, Seahorse analysis revealed the metabolic alterations induced by NR treatment that align with these earlier findings. Our findings indicated that NR treatment enhanced T_CM_ differentiation *in vitro*, and these NR-treated T cells exhibited superior anti-tumor effects *in vivo*.

The Foxo signaling pathway, including Foxo1, Foxo3, Foxo4, and Foxo6, has been the focus of research for nearly two decades.^40^ Among these proteins, Foxo1 is notably important in T cells for regulating differentiation, trafficking, survival, and memory T cell responses.^40^ Our study found that CD8^+^ T cells treated with NR exhibited higher levels of Foxo1 protein and mRNA, as well as increased expression of Foxo1-targeted key transcriptional regulators associated with the memory phenotype (*Tcf7*, *Il7r*, *Lef1*, *Ccr7*, *Klf2*, *S1P1*, and *Cxcr4*).^41^^,^^42^^,^^43^ While no difference in the gene expression of *Sell* was observed, the expression of the *Sell*-coding protein L-selectin (CD62L) was significantly higher in the NR-treated CD8^+^ T cells, as confirmed by flow cytometry. These results suggest that boosting NAD^+^ levels by NR supplementation activates the Foxo1 signaling pathway in the CD8^+^ T cells. Our results align with prior findings that enhancing NAD^+^ levels via CD38 knockout can regulate mitochondrial fitness by upregulating Foxo1 activity.^15^ In addition, the benefits of NR on mitochondrial function and enhancement of T_CM_ differentiation were partially abolished by a Foxo1 inhibitor, indicating that Foxo1 contributed to these effects.

At present, only a few articles have investigated the effect of NR on T cells. Recent studies have demonstrated that NR regulates CD4^+^ T cell function, including reducing Th17 inflammation via NRF2 activation and arginine biosynthesis and suppressing Th17 while promoting regulatory T cell (Treg) differentiation.^44^^,^^45^ For CD8^+^ T cells, a recent study showed that under *in vitro* conditions mimicking the tumor microenvironment, NR supplementation prevented mitochondrial dysfunction.^22^ However, the effect of NR on CD8^+^ T cell fate and anti-tumor capacity under expansion culture conditions has yet to be clarified. In this study, we expanded CD8^+^ T cells in IL-2 to trigger T_EFF_/effector memory T cell (T_EM_) differentiation, as described in previous studies.^1^^,^^7^ Under IL-2 culture conditions, NR intervention enhanced T_CM_ properties in CD8^+^ T cells, including increased CD62L expression, upregulated memory-related gene expression, improved mitochondrial fitness and functions, and enhanced memory T cell responses, indicating that NR promoted T_CM_ differentiation. Notably, these NR-treated CD8^+^ T cells exhibited superior anti-tumor effects *in vitro* and *in vivo*. Our study suggested that the addition of NR to the T cell culture system during the *in vitro* expansion process should be considered in clinical settings, which may improve the efficacy of ACT.

To summarize, our data show that both *in vivo* and *in vitro* NR treatment can enhance the effectiveness of ACT, providing a rationale for NR as an adjuvant therapeutic strategy for ACT in the treatment of HCC. *In vitro* NR treatment improves mitochondrial fitness and functions, thereby promoting T_CM_ formation. These benefits may involve, at least in part, Foxo1-associated pathways. This finding suggests that NR has the potential to enhance the therapeutic efficacy of ACT by modulating mitochondrial fitness and T cell differentiation.

## Materials and methods

### Cell culture and reagents

Huh7 cells (human liver cancer cell line) were obtained from the National Collection of Authenticated Cell Cultures. Huh7 cells expressing luciferase and EGFP (Huh7-GL cells) were derived from Huh7 cells and generated by ultra-purified lentivirus transduction (EX-hLUC-Lv201, Genecopoeia) following the manufacturer’s protocol. Hepa1-6 OVA cells were derived from the Hepa1-6 cell line (mouse liver cancer cell line). The establishment of the Hepa1-6 OVA cell line was described previously.^46^ Cells were cultured in Dulbecco’s modified Eagle’s medium (DMEM) (Gibco) containing 10% fetal bovine serum (FBS) (NEWZERUM) and 1% penicillin-streptomycin (Gibco). Cells were maintained at 37°C in a humidified, 5% CO_2_ atmosphere. NR chloride was from ChromaDex. AS1842856 was purchased from MedChemExpress.

### Mice

C57BL/6-Tg(TcraTcrb)1100Mjb/J mice (OT-I mice) and NOD.Cg-P*rkdc*^*scid*^ *Il2rg*^*tm1Wjl*^/SzJ mice (NSG mice) were from the Jackson Laboratory. C57BL/6J mice and BALB/c nu/nu mice were purchased from Gempharmatech (Guangzhou, China). Mice were housed in specific pathogen-free (SPF) animal research facilities. In consideration of animal welfare, mice were euthanized after rapid weight loss of 15%–20% within a few days or when they exhibited distress.

### Establishment of GPC3-specific CAR-T cells

To generate GPC3-specific CAR-T cells, the GPC3-specific single-chain variable fragment (anti-GPC3 scFv) was cloned into lentiviral vectors expressing the CD8 leader, CD8 hinge, CD28 transmembrane region (CD28 TM), CD28 and 4-1BB co-stimulation region, CD3ζ signaling domain, and EGFP. The lentiviral vector plasmid encoding the anti-GPC3 CAR sequence was synthesized and packaged by GenScript (Nanjing, China).

Human T cells were isolated from peripheral blood mononuclear cells (PBMCs) of healthy donors. PBMCs were separated by density gradient centrifugation using the Human Peripheral Blood Lymphocyte Separation Medium (TBD). Then, primary human T cells were purified by positive selection using CD3^+^ Microbeads (Miltenyi Biotec) or negative selection with the EasySep Human T cell Isolation Kit (STEMCELL). Human T cells were cultured in RPMI-1640 supplemented with 10% FBS, GlutaMAX (Gibco), β-mercaptoethanol (Gibco), sodium pyruvate (Gibco), and 10 ng/mL recombinant human IL-2 (Peprotech). T cells were stimulated with anti-CD3/CD28 microbeads (Miltenyi Biotec) for 48 h. After activation, T cells were transduced with lentiviral vectors expressing anti-GPC3 CAR. After transduction, anti-GPC3 CAR-T cells were cultured in a fresh medium with rhIL-2. Three days post-transfection, anti-GPC3 CAR-T cells were purified by fluorescence-activated cell sorting (FACS).

### *In vivo* models with adoptive anti-GPC3 CAR-T cell therapy

For the subcutaneous cell-derived xenograft (CDX) tumor model, NSG mice were subcutaneously injected with 2 × 10^6^ Huh-7 cells into the right rear flanks on day 0. Tumor volume was measured with a digital caliper every 3 days and calculated as length × width^2^ × 0.5. After 2 weeks, mice were randomly sorted into four groups (*n* = 5/group): control (CON), NR treatment (NR), anti-GPC3 CAR-T cell therapy (CAR-T), and anti-GPC3 CAR-T cell therapy plus NR treatment (CAR-T + NR). On day 15, mice in CAR-T/CAR-T + NR groups received 1 × 10^7^ anti-GPC3 CAR-T cells via tail vein injection, while those in CON/NR groups received an equivalent vehicle volume. The next day, NR and CAR-T + NR groups were given 400 mg/kg NR daily by gavage, and the others received vehicle.

For the metastatic tumor model, a total of 2 × 10^6^ Huh-7 cells were intraperitoneally (i.p.) injected into NSG mice. After 2 weeks, mice were randomly sorted into CON, NR, CAR-T, or CAR-T + NR groups (*n* = 5/group) and received corresponding therapy. On days 14, 21, and 35, mice were i.p. injected with 150 mg/kg D-luciferin (MCE), followed by imaging using an In Vivo Imaging System (IVIS; PerkinElmer). Quantification of bioluminescence was performed with Living Image Software (PerkinElmer).

### Isolation, activation, and expansion of OT-I CD8^+^ T cells

Mouse CD8^+^ T cells were isolated from the spleen of 6- to 18-week-old OT-I mice and purified via negative selection using the CD8a^+^ T cell Isolation Kit (Miltenyi Biotec). The purity of CD8^+^ T cells exceeded 95%. OT-I CD8^+^ T cells were activated by 10 ng/mL OVA_257-264_ (SIINFEKL) peptide (MedChemExpress) for 72 h. After activation, OT-I CD8^+^ T cells were cultured in RPMI-1640 supplemented with 10% FBS, GlutaMAX (Gibco), β-mercaptoethanol (Gibco), sodium pyruvate (Gibco), and 100 U/mL recombinant murine IL-2 (Peprotech). NR administration was initiated on day 0 with a final concentration of 500 μmol/L in a T cell culture medium.

### *In vivo* models with adoptive OT-I CD8^+^ T cell therapy

For the subcutaneous xenograft tumor models, 5 × 10^6^ Hepa1-6 OVA cells were subcutaneously injected into the right rear flanks of BALB/c nude mice. Tumor volume was measured every 3 days. After 6–7 days, mice were randomly allocated into three groups (*n* = 6–9/group): PBS, CON, and NR. Non-NR-treated and NR-treated day-7 OT-I CD8^+^ T cells were transferred into mice of the CON and NR groups via tail vein, respectively, while the PBS group received an equivalent volume of saline vehicle. For the weekly ACT model, mice were randomly sorted into two groups (*n* = 5/group): CON and NR. OT-I CD8^+^ T cells were intravenously transferred into tumor-bearing mice on days 6 and 14. Mice were imaged using the IVIS before sacrifice.

For the hematogenous metastatic tumor model, 2 × 10^6^ Hepa1-6 OVA cells were injected into the tail vein of nude mice. *In vivo* bioluminescent imaging was performed every 2 weeks. On day 8 after inoculation, mice were randomly sorted into the PBS, CON, or NR group (*n* = 6–8/group) and received the corresponding therapy.

### *In vitro* killing assay

Target cells (Hepa1-6 OVA cells) were co-cultured with *in*-*vitro*-cultured day-7 OT-I CD8^+^ T cells at the indicated E:T ratio in 3–4 technical replicate wells of white 96-well plates for 24 h. The target cell viability was evaluated by adding 300 μg/mL D-Luciferin and detecting the luminescence signal using a multi-mode microplate reader (BioTek Synergy H1, Agilent). The luminescence signal of non-co-culture target cells was considered as a reference. Values were expressed as the percentage of killing (%) calculated as (1 − experimental signal/reference signal) × 100.

### Flow cytometry

*In*-*vitro*-cultured OT-I CD8^+^ T cells or human anti-GPC3 CAR-T cells were harvested and resuspended in PBS supplemented with 2% FBS (FACS buffer). For surface marker staining, cells were incubated with fluorophore-labeled primary antibodies and subsequently stained with 7-AAD or propidium iodide (PI) viability staining solution (eBioscience) prior to detection. For intercellular staining, cells were pretreated with protein transport inhibitor cocktail (eBioscience) for 6–8 h before harvest. Cells were labeled with Fixable Viability Dye eFluor 780 (eBioscience) to exclude dead cells, then fixed and permeabilized using an Intracellular Fixation & Permeabilization Buffer Set (eBioscience) and stained with primary antibodies for intracellular protein. For mitochondrial ROS detection, cells were stained with the mitoSOX Red Kit (Beyotime) following the manufacturer’s protocol. For the CFSE cell proliferation assay, cells were labeled with CFSE dye (eBioscience) following the manufacturer’s protocol and analyzed after 3 days. For apoptosis detection, cells were stained with Annexin V Apoptosis Detection Kits (eBioscience) following the manufacturer’s protocol. All samples were detected using a CytoFlex/CytoFlex S flow cytometer (Beckman Coulter) or LSRFortessa X-20 (BD BioSciences). Data were analyzed using FlowJo or CytExpert 2.3 software. The antibodies used in flow cytometry included: PE anti-mouse CD3ε (17A2, BioLegend, 100205), APC anti-mouse CD8α (53–6.7, BioLegend, 100711), PE anti-mouse CD62L (MEL-14, eBioscience, 12-0621-81), APC anti-mouse/human CD44 (IM7, eBioscience, 17-0441-81), eFluor 450 anti-mouse TNF-α (MP6-XT22, eBioscience, 48-7821-80), PE anti-mouse granzyme B (NGZB, eBioscience, 12-8898-80), APC anti-mouse IFN-γ (XMG1.2, eBioscience, 17-7311-81), APC anti-mouse perforin (S16009B, BioLegend, 154403), Brilliant Violet 605 anti-mouse/human KLRG1 (2F1/KLRG1, BioLegend, 138419), eFluor 660 anti-mouse/human T-bet (eBio4B10, eBioscience, 50-5825-82), PE anti-human CD62L (DREG56, eBioscience, 12-0629-41), BV750 anti-human CD45RO (UCHL1, BD OptiBuild, 746942), Alexa Fluor 647 anti-human LAG-3/CD223 (T47-530, BD Pharmingen, 565716), BV421 anti-human Tim-3/CD366 (7D3, BD Horizon, 565562), BB660 anti-human PD-1/CD279 (MIH4, BD Biosciences, 624295), APC anti-human IFN-γ (4 S.B3, BioLegend, 502511), Brilliant Violet 421 anti-human perforin (dG9, BioLegend, 308121), BV750 anti-human TNF (MAb11, BD Horizon, 566359), and PE anti-human/mouse granzyme B (QA16A02, BioLegend, 372207).

### MMP detection

MMP was monitored by JC-1 dye staining (Beyotime) and detected by flow cytometry or CLSM. The red (aggregates) to green (monomer) fluorescence intensity ratio was used to evaluate mitochondrial depolarization, expressed as a fold change relative to the control group.

### Measurement of NAD^+^ level

The intracellular NAD^+^ content of *in*-*vitro*-cultured OT-I CD8^+^ T cells was detected using the NAD^+^/NADH Assay Kit with WST-8 (Beyotime). The amounts of total NAD^+^/NADH (NAD_total_) and NADH were detected by a microplate reader at 450 nm. The NAD^+^ content was calculated as NAD_total_ − NADH. All data were normalized by total protein concentration.

### Seahorse extracellular flux assay

The mitochondrial respiration and glycolysis of *in*-*vitro*-cultured OT-I CD8^+^ T cells were analyzed using a Seahorse XFe 96 analyzer (Agilent). Seahorse cell culture microplates were pre-coated with 22.4 μg/mL Cell-Tak (Corning) solution to enhance cell adhesion. OT-I CD8^+^ T cells were seeded at 1.8 × 10^5^ cells per well. Mitochondrial function was evaluated by measuring the OCR using the Seahorse XF Cell Mito Stress Test Kit (Agilent). 1.5 μmol/L oligomycin, 2.0 μmol/L FCCP, and 0.5 μmol/L rotenone/antimycin were injected at the indicated times. Glycolytic function was evaluated by measuring the ECAR with the Seahorse XF Glycolysis Stress Test Kit (Agilent). Seahorse data were collected by Wave 2.6.1 software (Agilent), automatically calculated by the Seahorse XF Report Generator, and exported to an Excel or GraphPad file.

### Western blot

*In*-*vitro*-cultured OT-I CD8^+^ T cells were harvested, washed, and lysed with RIPA buffer (Beyotime). Total protein concentrations were measured with a BCA protein assay kit (Beyotime). The primary antibodies used in western blot included anti-Foxo1 (Cell Signaling Technology, #2880), anti-TOM20 (Cell Signaling Technology, #42406), anti-PGC-1α (Cell Signaling Technology, #2178), anti-CD8α (Abcam, ab209775), anti-Tubulin (Abcam, ab6160), anti-β-actin (Santa Cruz Biotechnology, sc-47778), anti-IFN-γ (Zen Bio, R381656), anti-TNF-α (Zen Bio, 346654), and anti-granzyme B (Zen Bio, 252579). Secondary antibodies were purchased from Jackson ImmunoResearch Laboratories.

### Quantification of mRNA

Total RNA of *in*-*vitro*-cultured OT-I CD8^+^ T cells was extracted using TRIzol Reagent (Invitrogen) and reverse transcribed using the Prime Script RT Reagent Kit with gDNA Eraser (TAKARA) following the manufacturer’s protocol. Quantitative real-time PCR measurements were performed with ABI ViiATM7Dx or QuantStudio 6 Pro (Thermo Fisher Scientific) using the TB Green Premix Ex Raq II (Tli RNaseH Plus) Kit (TAKARA). Relative mRNA expression of target genes was calculated by the 2^(−ΔΔ*Cq*)^ method with *Actb* as endogenous control. Primers used in this study are listed in Table S1.

### RNA-seq analysis

Total RNA of *in*-*vitro*-cultured OT-I CD8^+^ T cells was extracted using TRIzol Reagent and resuspended in RNase-free water. RNA-seq samples were sequenced on the BGISEQ platform. Raw data were filtered using SOAPnuke 1.5.2 software (BGI Genomics) and aligned to the *Mus musculus* genome assembly GRCm39 with HISAT2.0.4 software (BGI Genomics). The detection of differential genes was determined using the DEseq2 method with a *q* value (adjusted *p* value) ≤ 0.05. For enrichment analysis, pathways with a *q* value ≤ 0.05 were significantly enriched among the differentially expressed genes (DEGs).

### Statistical analysis

Results were presented as mean ± standard error of the mean (SEM). Data were analyzed using GraphPad Prism 10 software. Statistical analysis methods are described in the figure legends. *p* < 0.05 was considered statistically significant.

### Ethics approval

All animal experiments in this study were approved by the Animal Care and Protection Committee of Sun Yat-sen University (No. 2023000374 and 2023001184). All human sample collections in this study were approved by the Institutional Review Board of the Second Affiliated Hospital of the Guangzhou Medical University (2017-hs-04). The informed consent was obtained from participants prior to recruitment.

## Data and code availability

All data supporting the findings of this study are available from the corresponding author upon request.

## Acknowledgments

We thank Prof. Meng Li from the Fourth Military Medical University for generously providing us with the Hepa1-6 OVA cells. This work was supported by the 10.13039/501100001809National Natural Science Foundation of China (nos. 81872613 to L.Y. and 82473311 and 81872069 to Z.Z.) and the 10.13039/501100010256Guangzhou Municipal Science and Technology Project (no. 2023B03J0067 to Z.Z.).

## Author contributions

L.Y. and Z.Z. conceived and designed the study. N.P., J.H., and Y.X. conducted the animal experiments. N.P., L.P., and Y.X. performed the cell experiments. C.Z. collected human HCC tumor tissues, and C.Z. and N.P. performed and analyzed immunohistochemical staining. N.P., Y.X., J.H., C.Z., Y.G., L.P., X.L., M.C., Q.X., and X.W. participated in data analysis, including both animal and cell experiments. L.Y. supervised the execution of this study and reviewed all experimental data. N.P. wrote the manuscript, which was subsequently revised by L.Y. and Z.Z. All authors read and approved the final manuscript.

## Declaration of interests

The authors declare no competing interests.

## Supplemental information

Document S1. Figures S1–S8 and Table S1

Document S2. Article plus supplemental information

## References

[1] Klebanoff C.A., Gattinoni L., Torabi-Parizi P., Kerstann K., Cardones A.R., Finkelstein S.E., Palmer D.C., Antony P.A., Hwang S.T., Rosenberg S.A. (2005). Central memory self/tumor-reactive CD8+ T cells confer superior antitumor immunity compared with effector memory T cells. Proc. Natl. Acad. Sci. USA.

[2] Sukumar M., Kishton R.J., Restifo N.P. (2017). Metabolic reprograming of anti-tumor immunity. Curr. Opin. Immunol..

[3] Han J., Khatwani N., Searles T.G., Turk M.J., Angeles C.V. (2020). Memory CD8(+) T cell responses to cancer. Semin. Immunol..

[4] Riera-Domingo C., Audigé A., Granja S., Cheng W.C., Ho P.C., Baltazar F., Stockmann C., Mazzone M. (2020). Immunity, Hypoxia, and Metabolism-the Menage a Trois of Cancer: Implications for Immunotherapy. Physiol. Rev..

[5] O'Sullivan D., Pearce E.L. (2015). Targeting T cell metabolism for therapy. Trends Immunol..

[6] MacIver N.J., Michalek R.D., Rathmell J.C. (2013). Metabolic regulation of T lymphocytes. Annu. Rev. Immunol..

[7] Buck M.D., O’Sullivan D., Klein Geltink R.I., Curtis J.D., Chang C.H., Sanin D.E., Qiu J., Kretz O., Braas D., van der Windt G.W. (2016). Mitochondrial Dynamics Controls T Cell Fate through Metabolic Programming. Cell.

[8] Kishton R.J., Sukumar M., Restifo N.P. (2017). Metabolic Regulation of T Cell Longevity and Function in Tumor Immunotherapy. Cell Metab..

[9] Sukumar M., Liu J., Ji Y., Subramanian M., Crompton J.G., Yu Z., Roychoudhuri R., Palmer D.C., Muranski P., Karoly E.D. (2013). Inhibiting glycolytic metabolism enhances CD8+ T cell memory and antitumor function. J. Clin. Invest..

[10] Xie N., Zhang L., Gao W., Huang C., Huber P.E., Zhou X., Li C., Shen G., Zou B. (2020). NAD(+) metabolism: pathophysiologic mechanisms and therapeutic potential. Signal. Transduct. Target. Ther..

[11] Amjad S., Nisar S., Bhat A.A., Shah A.R., Frenneaux M.P., Fakhro K., Haris M., Reddy R., Patay Z., Baur J., Bagga P. (2021). Role of NAD(+) in regulating cellular and metabolic signaling pathways. Mol. Metab..

[12] Chatterjee S., Chakraborty P., Mehrotra S. (2019). CD38-NAD(+)-Sirt1 axis in T cell immunotherapy. Aging (Albany NY).

[13] Wang Y., Wang F., Wang L., Qiu S., Yao Y., Yan C., Xiong X., Chen X., Ji Q., Cao J. (2021). NAD(+) supplement potentiates tumor-killing function by rescuing defective TUB-mediated NAMPT transcription in tumor-infiltrated T cells. Cell Rep..

[14] Gerner R.R., Macheiner S., Reider S., Siegmund K., Grabherr F., Mayr L., Texler B., Moser P., Effenberger M., Schwaighofer H. (2020). Targeting NAD immunometabolism limits severe graft-versus-host disease and has potent antileukemic activity. Leukemia.

[15] Chatterjee S., Daenthanasanmak A., Chakraborty P., Wyatt M.W., Dhar P., Selvam S.P., Fu J., Zhang J., Nguyen H., Kang I. (2018). CD38-NAD(+)Axis Regulates Immunotherapeutic Anti-Tumor T Cell Response. Cel Metab..

[16] Bieganowski P., Brenner C. (2004). Discoveries of nicotinamide riboside as a nutrient and conserved NRK genes establish a Preiss-Handler independent route to NAD+ in fungi and humans. Cell.

[17] Bogan K.L., Brenner C. (2008). Nicotinic acid, nicotinamide, and nicotinamide riboside: a molecular evaluation of NAD+ precursor vitamins in human nutrition. Annu. Rev. Nutr..

[18] Zhang H., Ryu D., Wu Y., Gariani K., Wang X., Luan P., D’Amico D., Ropelle E.R., Lutolf M.P., Aebersold R. (2016). NAD^+^ repletion improves mitochondrial and stem cell function and enhances life span in mice. Science.

[19] Wang S., Wan T., Ye M., Qiu Y., Pei L., Jiang R., Pang N., Huang Y., Liang B., Ling W. (2018). Nicotinamide riboside attenuates alcohol induced liver injuries via activation of SirT1/PGC-1alpha/mitochondrial biosynthesis pathway. Redox Biol..

[20] Elhassan Y.S., Kluckova K., Fletcher R.S., Schmidt M.S., Garten A., Doig C.L., Cartwright D.M., Oakey L., Burley C.V., Jenkinson N. (2019). Nicotinamide Riboside Augments the Aged Human Skeletal Muscle NAD(+) Metabolome and Induces Transcriptomic and Anti-inflammatory Signatures. Cel Rep..

[21] Pang N., Hu Q., Zhou Y., Xiao Y., Li W., Ding Y., Chen Y., Ye M., Pei L., Li Q. (2023). Nicotinamide Adenine Dinucleotide Precursor Suppresses Hepatocellular Cancer Progression in Mice. Nutrients.

[22] Yu Y.R., Imrichova H., Wang H., Chao T., Xiao Z., Gao M., Rincon-Restrepo M., Franco F., Genolet R., Cheng W.C. (2020). Disturbed mitochondrial dynamics in CD8(+) TILs reinforce T cell exhaustion. Nat. Immunol..

[23] Lapatto H.A.K., Kuusela M., Heikkinen A., Muniandy M., van der Kolk B.W., Gopalakrishnan S., Pöllänen N., Sandvik M., Schmidt M.S., Heinonen S. (2023). Nicotinamide riboside improves muscle mitochondrial biogenesis, satellite cell differentiation, and gut microbiota in a twin study. Sci. Adv..

[24] Fang E.F., Kassahun H., Croteau D.L., Scheibye-Knudsen M., Marosi K., Lu H., Shamanna R.A., Kalyanasundaram S., Bollineni R.C., Wilson M.A. (2016). NAD+ Replenishment Improves Lifespan and Healthspan in Ataxia Telangiectasia Models via Mitophagy and DNA Repair. Cel Metab..

[25] Cantó C., Houtkooper R.H., Pirinen E., Youn D.Y., Oosterveer M.H., Cen Y., Fernandez-Marcos P.J., Yamamoto H., Andreux P.A., Cettour-Rose P. (2012). The NAD(+) precursor nicotinamide riboside enhances oxidative metabolism and protects against high-fat diet-induced obesity. Cel Metab..

[26] Navas L.E., Carnero A. (2021). NAD(+) metabolism, stemness, the immune response, and cancer. Signal. Transduct. Target. Ther..

[27] Yong J., Cai S., Zeng Z. (2023). Targeting NAD(+) metabolism: dual roles in cancer treatment. Front. Immunol..

[28] Jiang Y., Luo Z., Gong Y., Fu Y., Luo Y. (2023). NAD(+) supplementation limits triple-negative breast cancer metastasis via SIRT1-P66Shc signaling. Oncogene.

[29] Tummala K.S., Gomes A.L., Yilmaz M., Graña O., Bakiri L., Ruppen I., Ximénez-Embún P., Sheshappanavar V., Rodriguez-Justo M., Pisano D.G. (2014). Inhibition of de novo NAD(+) synthesis by oncogenic URI causes liver tumorigenesis through DNA damage. Cancer Cell.

[30] Beltrà M., Pöllänen N., Fornelli C., Tonttila K., Hsu M.Y., Zampieri S., Moletta L., Corrà S., Porporato P.E., Kivelä R. (2023). NAD(+) repletion with niacin counteracts cancer cachexia. Nat. Commun..

[31] Katsuyama E., Suarez-Fueyo A., Bradley S.J., Mizui M., Marin A.V., Mulki L., Krishfield S., Malavasi F., Yoon J., Sui S.J.H. (2020). The CD38/NAD/SIRTUIN1/EZH2 Axis Mitigates Cytotoxic CD8 T Cell Function and Identifies Patients with SLE Prone to Infections. Cel Rep..

[32] Magnone M., Bauer I., Poggi A., Mannino E., Sturla L., Brini M., Zocchi E., De Flora A., Nencioni A., Bruzzone S. (2012). NAD+ levels control Ca2+ store replenishment and mitogen-induced increase of cytosolic Ca2+ by Cyclic ADP-ribose-dependent TRPM2 channel gating in human T lymphocytes. J. Biol. Chem..

[33] Ye B., Pei Y., Wang L., Meng D., Zhang Y., Zou S., Li H., Liu J., Xie Z., Tian C. (2024). NAD(+) supplementation prevents STING-induced senescence in CD8(+) T cells by improving mitochondrial homeostasis. J. Cel. Biochem..

[34] Hope H.C., de Sostoa J., Ginefra P., Andreatta M., Chiang Y.H., Ronet C., Pich-Bavastro C., Corria Osorio J., Kuonen F., Auwerx J. (2025). Age-associated nicotinamide adenine dinucleotide decline drives CAR-T cell failure. Nat. Cancer.

[35] Jeng M.Y., Hull P.A., Fei M., Kwon H.S., Tsou C.L., Kasler H., Ng C.P., Gordon D.E., Johnson J., Krogan N. (2018). Metabolic reprogramming of human CD8(+) memory T cells through loss of SIRT1. J. Exp. Med..

[36] Guo Y., Xie Y.-Q., Gao M., Zhao Y., Franco F., Wenes M., Siddiqui I., Bevilacqua A., Wang H., Yang H. (2021). Metabolic reprogramming of terminally exhausted CD8+ T cells by IL-10 enhances anti-tumor immunity. Nat. Immunol..

[37] Vardhana S.A., Hwee M.A., Berisa M., Wells D.K., Yost K.E., King B., Smith M., Herrera P.S., Chang H.Y., Satpathy A.T. (2020). Impaired mitochondrial oxidative phosphorylation limits the self-renewal of T cells exposed to persistent antigen. Nat. Immunol..

[38] Berger C., Jensen M.C., Lansdorp P.M., Gough M., Elliott C., Riddell S.R. (2008). Adoptive transfer of effector CD8+ T cells derived from central memory cells establishes persistent T cell memory in primates. J. Clin. Invest..

[39] Buck M.D., O’Sullivan D., Pearce E.L. (2015). T cell metabolism drives immunity. J. Exp. Med..

[40] Luo C.T., Li M.O. (2018). Foxo transcription factors in T cell biology and tumor immunity. Semin. Cancer Biol..

[41] Kim M.V., Ouyang W., Liao W., Zhang M.Q., Li M.O. (2013). The transcription factor Foxo1 controls central-memory CD8+ T cell responses to infection. Immunity.

[42] Michelini R.H., Doedens A.L., Goldrath A.W., Hedrick S.M. (2013). Differentiation of CD8 memory T cells depends on Foxo1. J. Exp. Med..

[43] Arojo O.A., Ouyang X., Liu D., Meng T., Kaech S.M., Pereira J.P., Su B. (2018). Active mTORC2 Signaling in Naive T Cells Suppresses Bone Marrow Homing by Inhibiting CXCR4 Expression. J. Immunol..

[44] Han K., Singh K., Meadows A.M., Sharma R., Hassanzadeh S., Wu J., Goss-Holmes H., Huffstutler R.D., Teague H.L., Mehta N.N. (2023). Boosting NAD preferentially blunts Th17 inflammation via arginine biosynthesis and redox control in healthy and psoriasis subjects. Cell Rep. Med..

[45] Pan W., Tsokos M.G., Scherlinger M., Li W., Tsokos G.C. (2024). The PP2A regulatory subunit PPP2R2A controls NAD(+) biosynthesis to regulate T cell subset differentiation in systemic autoimmunity. Cel Rep..

[46] Wang Z., He L., Li W., Xu C., Zhang J., Wang D., Dou K., Zhuang R., Jin B., Zhang W. (2021). GDF15 induces immunosuppression via CD48 on regulatory T cells in hepatocellular carcinoma. J. Immunother. Cancer.

