# Supplementary material for: Nicotinamide riboside enhances adoptive T cell therapy by promoting memory differentiation and metabolic fitness
Source: Mol Ther. 2026 Jun 2;34(8):4743–57. doi: 10.1016/j.ymthe.2026.05.026 (PMC13464305; doi:10.1016/j.ymthe.2026.05.026)
Supplement: Supplementary file 1 [file mmc1.pdf]

YMTHE, Volume *34*

Supplemental Information

Nicotinamide riboside enhances adoptive T cell therapy by promoting memory differentiation and metabolic fitness

Nengzhi Pang, Jinping He, Lei Pei, Ying Xiao, Yingying Gu, Cheng Zhi, Xuye Lai, Mingtao Chen, Qiyao Xiao, Xueqian Wu, Zhenfeng Zhang, and Lili Yang

Table S1 Primers used in Quantitative real-time PCR

Gene	Forward Primer	Reverse Primer
Foxo1	GGGTCCCACAGCAACGATG	CACCAGGGAATGCACGTCC
Tcf7	CCCTCAATGCGTTCATGCTTT	CTTGCGGGCCAGTTCATAGT
Ccr7	ATGGACCCAGGTGTGCTTCT	GGCAGGAACCAGGCCTTAAA
Actb	GTGGTGGTGAAGCTGTAGCC	AGCCATGTACGTAGCCATCC

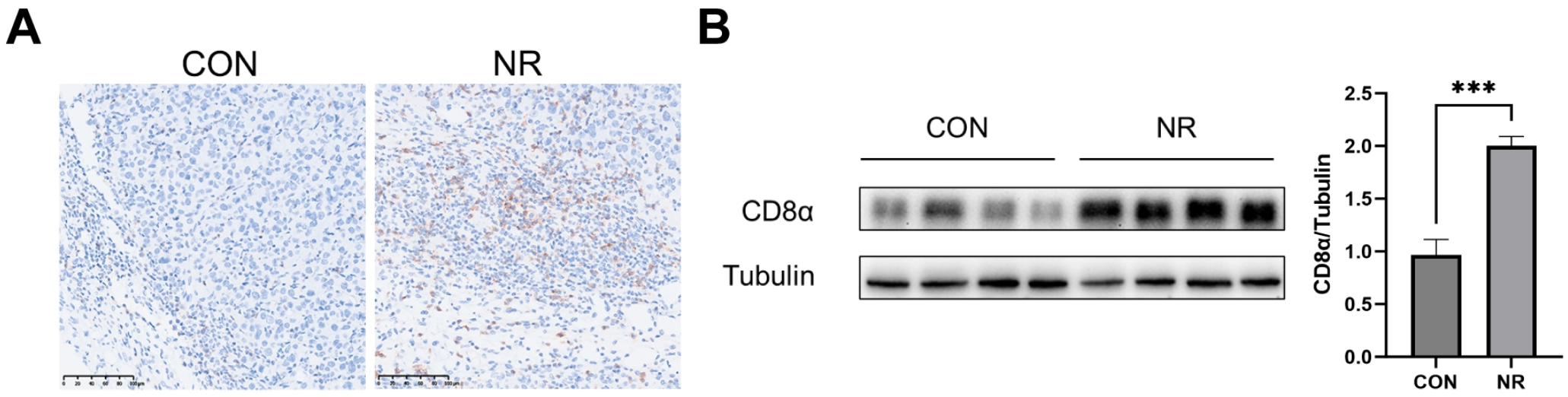

Fig. S1 NR increased the CD8α expression in tumor tissues. CD8α expression in tumor tissues of the subcutaneous xenograft tumor model established by Hepa1-6 cells in C57BL/6J mice. NR treatment (400 mg/kg body weight) was administrated in gavage daily for 2 weeks. ***P<0.001; P-value was calculated using Unpaired t test (B).

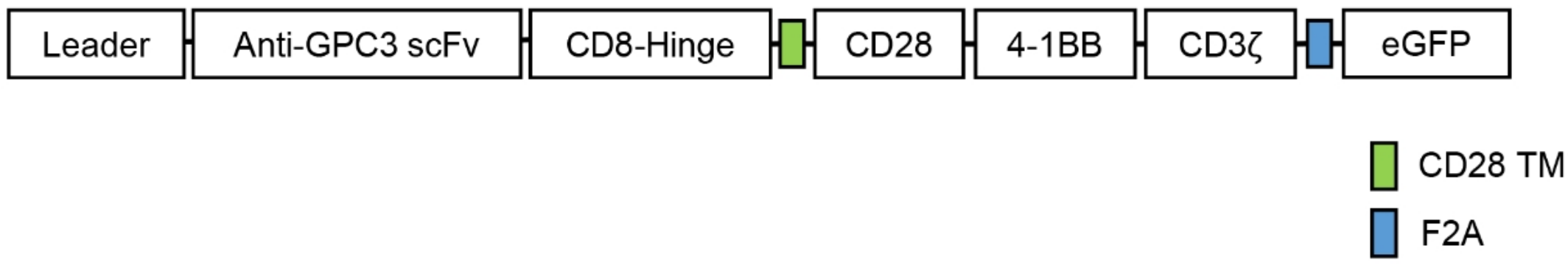

Fig. S2 Schematic of the lentiviral vector of anti-GPC3 CAR.

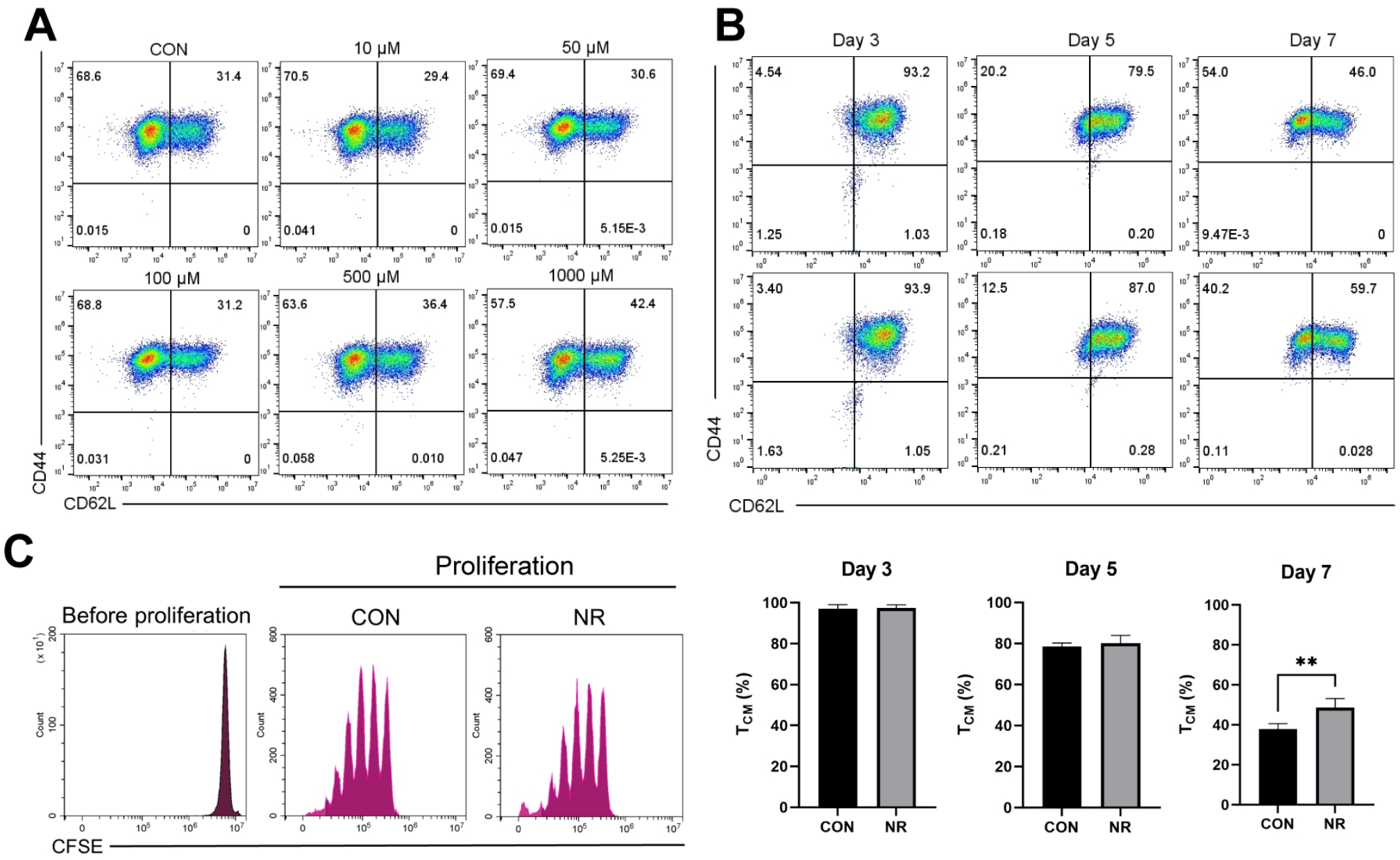

Fig. S3 NR enhanced the viability of CD8+ T cells. (A-B). Surface expression of CD44 and CD62L on CD8+ T cells treated with different concentrations of NR for the indicated time. **(C).** CFSE cell proliferation assay. ** *P*<0.01; *P*-values were calculated using Paired t test.

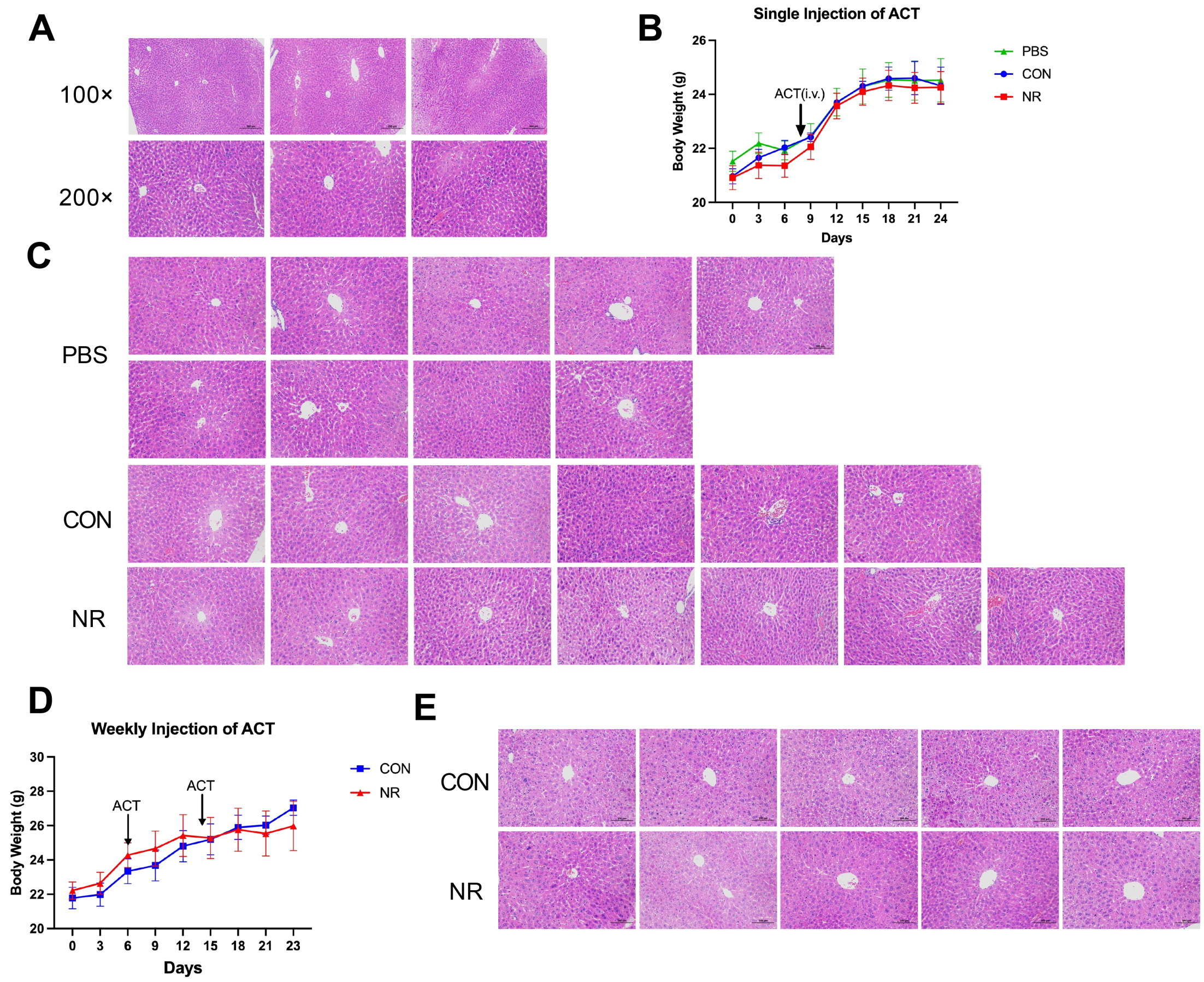

Fig. S4 Histological and body weight analysis of NR-treated ACT models, related to Figure 3. (A) Liver H&E from healthy mice, scale bars: 200 μm (top) and 100 μm (bottom). (B-C) Single injection of ACT model: body weight changes and liver H&E staining, Scale bars: 100 μm. (D-E) Weekly injection of ACT model: body weight changes and liver H&E staining, Scale bars: 100 μm.

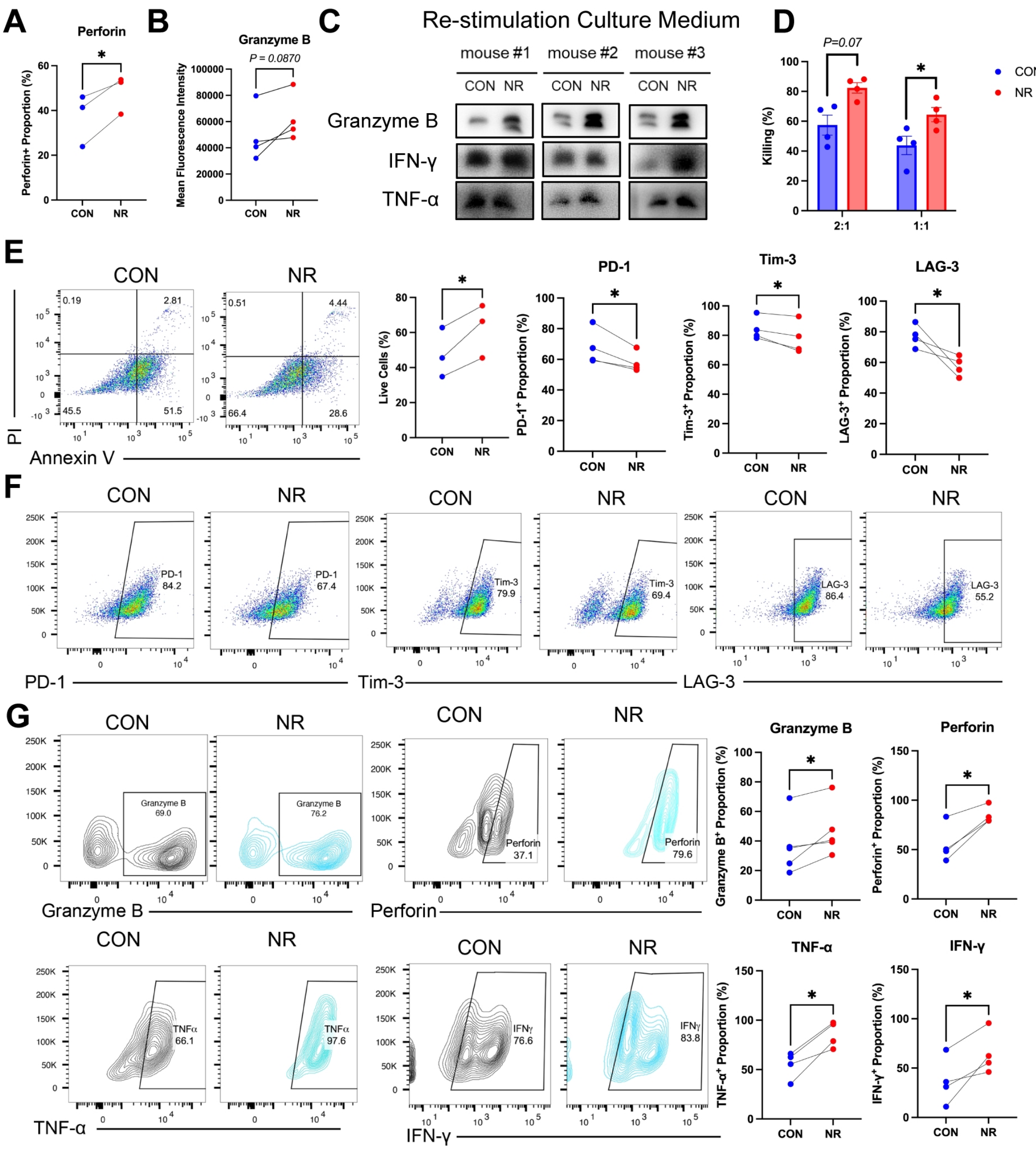

Fig. S5 NR-treated CD8$^+$ T cells showed a stronger recall response. (A-B). Quantification of FC analysis of intracellular cytokines. *n*= 4 independent experiments. **(C).** Western blot of Granzyme B, IFNγ, and TNFα in the culture medium. *n* = 3 independent experiments. **(D).** In vitro cytotoxicity of anti-GPC3 CAR-T cells at 2:1 and 1:1 E:T ratios. *n* = 4 independent experiments. **(E-G).** After initial activation and 5-6 days of antigen-free rest, human anti-GPC3 CAR-T cells were rechallenged with GPC3$^+$ tumor cells for 48 hours. NR-treated CAR-T cells exhibited (E) lower apoptosis and higher cell viability, (F) downregulation of an

exhaustion marker, and (G) increased production of effector cytokines. n = 3-5 independent experiments. *$P < 0.05$; P-values were calculated using Paired t test.

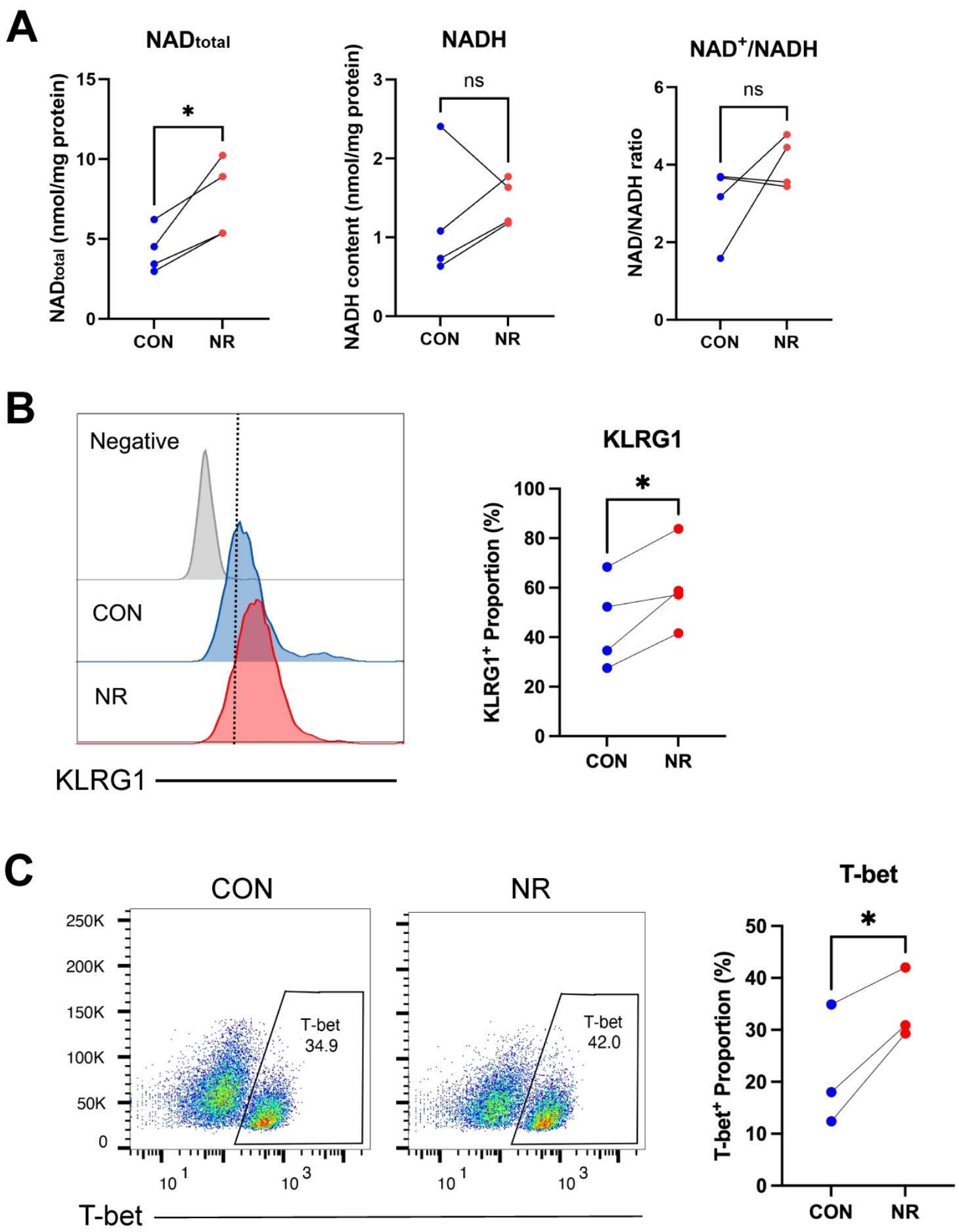

Fig. S6 NR boosted NAD$^+$ content in OT-I CD8$^+$ T cells. (A). Total NAD/NADH, NADH, and NAD$^+$/NADH ratio in T cells. *n*= 4 independent experiments. **(B-C).** Representative image and quantification of FC analysis of T cell terminal effector markers. *n*= 3-4 independent experiments. *$P < 0.05$; *P*-values were calculated using Paired t test.

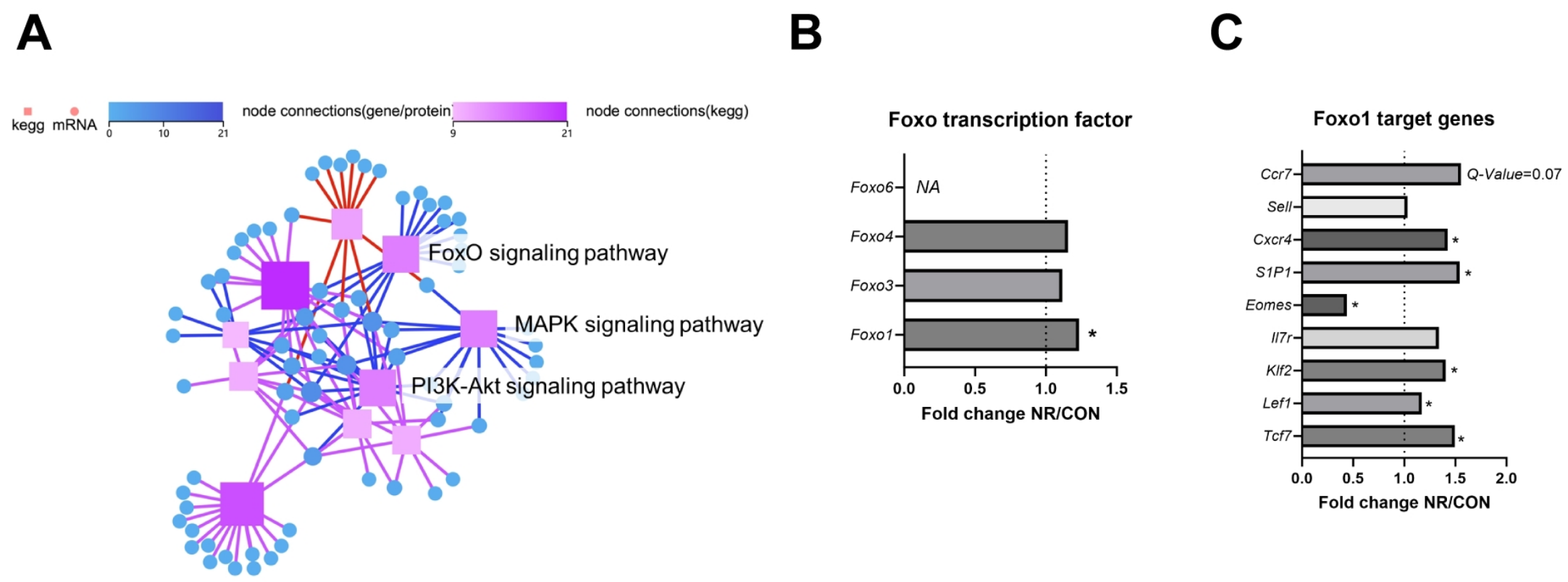

Fig. S7 NR regulated mitochondrial fitness via activating the Foxo1 signaling pathway. (A). KEGG pathway network analysis. **(B).** Four Foxo transcription factor genes expression in day-7 OT-I CD8$^+$ T cells. *NA,* not available; asterisk indicated differentially expressed genes (DEGs). **(C)**. Foxo1 target genes expression in day-7 OT-I CD8$^+$ T cells. Asterisk indicated differentially expressed genes (DEGs).

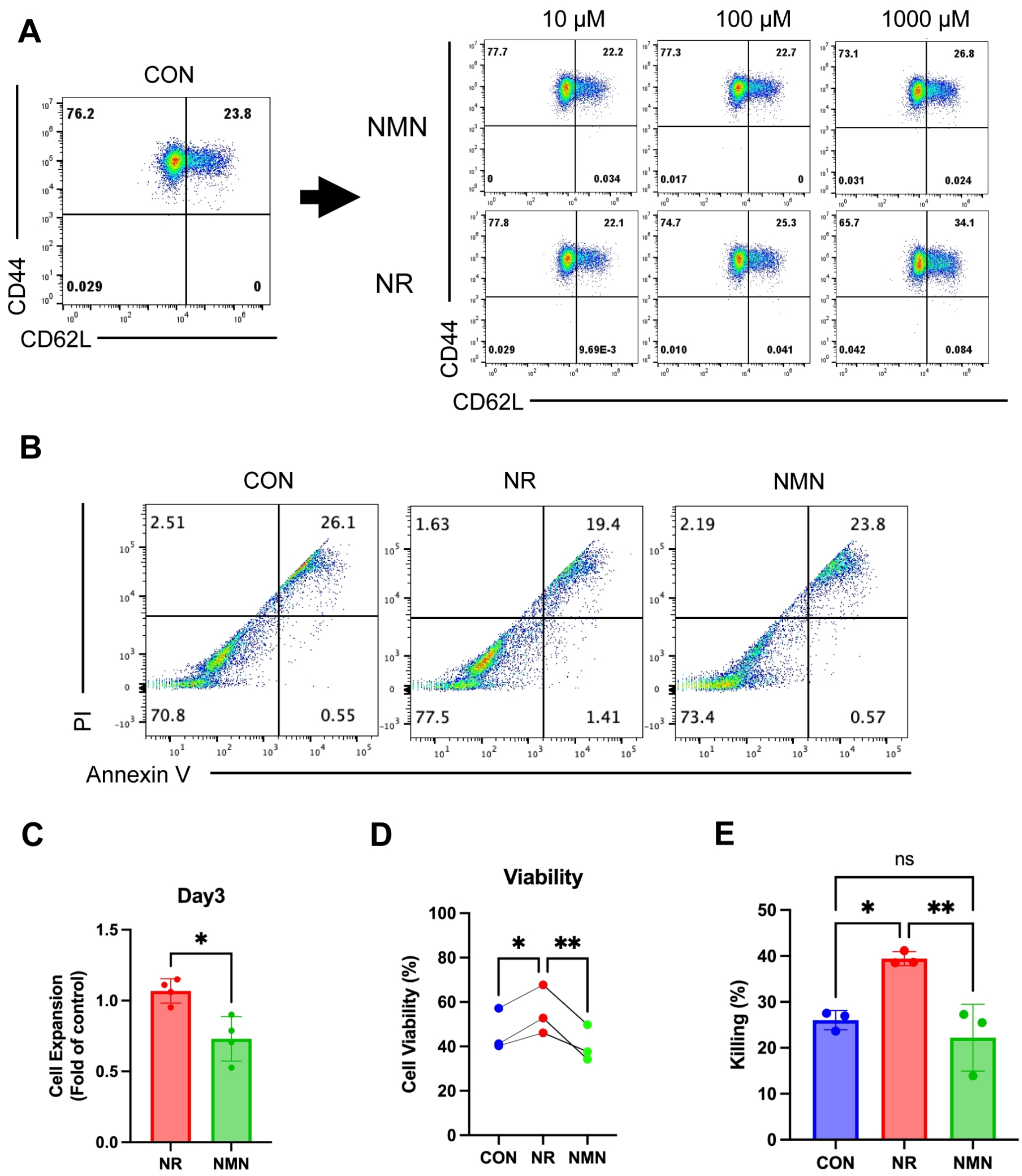

Fig. S8 Direct comparison of NR and NMN effects on CD8⁺ T cell. (A). Surface expression of CD44 and CD62L on CD8⁺ T cells treated with different NAD⁺ precursors for 96 hours. **(B).** Apoptosis analysis by Annexin V/PI staining at day 7. **(C).** Cell expansion at day 3. **(D).** Cell viability at day 7. **(E).** In vitro killing activity at day 7 (E:T ratio = 1:1). ns, not significant, **P*< 0.05, ***P*< 0.01; *P*-values were calculated using Unpaired *t* test (C) and One-way ANOVA and Tukey's multiple comparisons test (D and E).
